# Genome-based taxonomy of *Burkholderia sensu lato:* Distinguishing closely related species

**DOI:** 10.1590/1678-4685-GMB-2023-0122

**Published:** 2023-11-03

**Authors:** Evelise Bach, Camila Gazolla Volpiano, Fernando Hayashi Sant’Anna, Luciane Maria Pereira Passaglia

**Affiliations:** 1 Instituto de Biociências, Departamento de Genética and Programa de Pós-Graduação em Genética e Biologia Molecular, Porto Alegre, RS, Brazil.; 2 Hospital Moinhos de Vento, Programa de Apoio ao Desenvolvimento Institucional do Sistema Único de Saúde (PROADI - SUS), Porto Alegre, RS, Brazil.

**Keywords:** Average Nucleotide Identity, Burkholderia, Paraburkholderia, Digital DNA-DNA hybridization, heterotypic synonyms

## Abstract

The taxonomy of *Burkholderia sensu lato* (*s.l.*) has been revisited using genome-based tools, which have helped differentiate closely related species. Many species from this group are indistinguishable through phenotypic traits and 16S rRNA gene sequence analysis. Furthermore, they also exhibit whole-genome Average Nucleotide Identity (ANI) values in the twilight zone for species circumscription (95-96%), which may impair their correct classification. In this work, we provided an updated *Burkholderia s.l.* taxonomy focusing on closely related species and give other recommendations for those developing genome-based taxonomy studies. We showed that a combination of ANI and digital DNA-DNA hybridization (dDDH) applying the universal cutoff values of 95% and 70%, respectively, successfully discriminates *Burkholderia s.l.* species. Using genome metrics with this pragmatic criterion, we demonstrated that i) *Paraburkholderia insulsa* should be considered a later heterotypic synonym of *Paraburkholderia fungorum;* ii) *Paraburkholderia steynii* differs from *P. terrae* by harboring symbiotic genes; iii) some *Paraburkholderia* are indeed different species based on dDDH values, albeit sharing ANI values close to 95%; iv) some *Burkholderia s.l.* indeed represent new species from the genomic viewpoint; iv) some genome sequences should be evaluated with care due to quality concerns.

## Introduction

In 1973, Palleroni et al. (1973) carried out ribosomal ribonucleic acid (rRNA)-DNA hybridization studies that indicated that the *Pseudomonas* genus was composed of five RNA homology groups (I-V). In 1992, Yabuuchi et al. (1992) proposed the creation of the genus *Burkholderia* for the RNA homology group II based on the 16S rRNA gene sequence, DNA-DNA hybridization (DDH) values, phenotypic characteristics, and composition of cellular lipids and fatty acids. Twelve years later, with as little as 34 validly described species, *Burkholderia* already exhibited a complex taxonomy. Due to resolution limitations of the 16S rRNA gene sequence analysis, Payne et al. (2005) developed a *recA* gene-based identification for the genus. Further multilocus sequence analysis (MLSA) indicated the presence of at least two distinct lineages within the genus (Estrada-de Los Santos et al., 2013), which corroborated several previous works based on 16S rRNA gene sequences (Gyaneshwar et al., 2011). Applying phylogenomics and analyzing 42 conserved molecular markers of sequence insertions or deletions (CSIs), Sawana et al. (2014) confirmed the presence of at least two different clades within *Burkholderia*. These authors proposed the division of the genus and the creation of the genus *Paraburkholderia*. 

The increasing availability of genomic data allowed the recognition of differential CSIs and the reclassification of some *Paraburkholderia* species to the new genus *Caballeronia* (Dobritsa and Samadpour, 2016). In addition, these authors provided the emended description of several species. In all these studies, *Burkholderia andropogonis* consistently formed a distinct clade. Through a combination of phylogeny based on 30 conserved genes and genome metrics, the evolutionary distance of *B. andropogonis* led to the creation of the monotypic genus *Robbsia* (Lopes-Santos et al., 2017). More recently, phylogenomics associated with Average Nucleotide Identity (ANI) calculations showed the necessity of additional divisions of the genus and the creation of the new genera *Mycetohabitans* and *Trinickia* (Estrada-de Los Santos et al., 2018). Moreover, polyphasic approaches led to the proposal of the novel *Pararobbsia* genus to accommodate two species, *Pararobbsia silviterrae* and *Pararobbsia alpina* comb. nov. (Lin et al., 2020). Therefore, *Burkholderia sensu lato* (*s.l.*) is currently composed of *Burkholderia sensu strictu* (*s.s.*), *Paraburkholderia, Caballeronia, Mycetohabitans, Trinickia, Robbsia,* and *Pararobbsia* species (Bach et al., 2022b).

Correctly identifying an isolate is helpful for many research areas since this information provides insights into the biotechnological potential, biosafety, clinical outcomes, ecological roles, and evolutionary origin of features. Accurate species assignment is especially critical for members of the *Burkholderia cepacia* complex (Bcc), which includes strains competent in producing a myriad of bioactive compounds with biotechnological applications but that may also cause worrisome lung infections in patients with cystic fibrosis (Bach et al., 2022a). Some pathogenic Bcc species exhibit higher patient-to-patient transmissibility and the disease caused by different species have distinct clinical outcomes (Pope et al., 2010). Another example within *Burkholderia s.s.* species are *Burkholderia mallei* and *Burkholderia pseudomallei,* which cause the zoonotic diseases glanders and melioidosis, respectively. While glanders is primarily a horse disease, melioidosis may affect humans and other animals (Godoy et al., 2003). Furthermore, distinct rhizobial *Paraburkholderia* species can establish a beneficial symbiotic relationship and form root nodules for nitrogen fixation in different legume species (Mavima et al., 2022). The species mentioned above are hardly distinguished by the evaluation of 16S rRNA or *recA* gene sequences, highlighting the importance of using genome metrics to differentiate closely related species.

Since 1987 the *ad hoc* committee of the International Committee for Systematic Bacteriology (current International Committee on Systematics of Prokaryotes, ICSP) agrees that the DNA sequence should be the reference standard to determine taxonomy (Wayne et al., 1987). At that time, the recommended procedure for defining a species were two measures of genetic relatedness: the change in the melting temperature (ΔTm) of heteroduplex DNA and the extent of DDH. Two strains should belong to the same species if presenting both 70% or more DDH relatedness and 5 ºC or less of ∆Tm. However, these procedures show significant technical drawbacks being prone to giving imprecise results (Goris et al., 2007; Sant’Anna et al., 2019). The genome metrics ANI and digital DDH (dDDH) are surrogates for the ΔTm and DDH, using the thresholds of 95 and 70%, respectively (Goris *et al.*, 2007; Meier-Kolthoff et al., 2013; Chun et al., 2018), providing more reliable and portable taxonomic results in substitution to wet-lab genomic relatedness comparisons. 

Another advantage of using genome metrics in taxonomy is adopting a universal cutoff value for species delimitation that defines an objective criterion for species circumscription and standardizes communication among the scientific community of different areas. However, different thresholds are suggested and might be accepted to delineate species that show diagnostic distinct phenotypic traits as is the case of closely related *Paraburkholderia* species (Mullins and Mahenthiralingam, 2021). Moreover, genome metrics are increasingly being used to split known species into novel ones (Velez et al., 2023) and detect the presence of synonyms, which are species described with different names that belong to the same species (Madhaiyan et al., 2022). These peculiarities and updates complicate the correct species assignment for those unfamiliar with the taxonomy of the group. In previous work, we studied the pangenome and provided a genome-based taxonomy of *Burkholderia s.l.* species (Bach et al., 2022b)*.* In this work, we provided an updated *Burkholderia s.l.* taxonomy focusing on closely related species to search for synonyms and give other recommendations for those developing genome-based taxonomy studies. 

## Material and Methods

*Burkholderiales* genome sequences were obtained from the RefSeq NCBI database in August 2022 and subjected to a cluster analysis (Table S1) to scan for synonyms. Briefly, the genome metrics were calculated with FastANI (Jain et al., 2018) and clustered with ProKlust (Volpiano et al., 2021), a graph-based approach for downstream analysis of large identity matrices. Genomes that formed clusters with ANI ≥ 95% were selected for further analysis.

Reference genome sequences of *Paraburkholderia* and selected *Burkholderia* and *Caballeronia* were downloaded from the RefSeq database up to March 2023 and quality was checked using CheckM (Parks et al., 2015) (Table S2). Genome metrics were calculated using FastANI (Jain et al., 2018), JSpecies (ANIb and ANIm), and Genome-to-Genome Distance Calculator (GGDC) web tools at http://jspecies.ribohost.com/jspeciesws/ and http://ggdc.dsmz.de/home.php, respectively. Two genomes were considered belonging to the same species if both metrics showed results above the thresholds recommended for species delineation. Phylogenomics was performed as described previously (Bach et al., 2022b). Briefly, genomes were annotated with Prokka (Seemann, 2014) and single-copy orthologous proteins were obtained by the intersection of results provided by three clustering algorithms implemented in the GET-HOMOLOGUES tool using default parameters (Contreras-Moreira and Vinuesa, 2013). The phylogeny was reconstructed following the GET-PHYLOMARKERS pipeline, using the maximum likelihood approach and estimating the best tree through IQ-TREE (Vinuesa *et al.*, 2018). 

## Results and Discussion

Our previous pangenome and phylogenomic study of *Burkholderia s.l*. has already indicated the presence of many new species and synonyms within the group (Bach et al., 2022b). Our work corroborated previous results regarding Bcc members (Jin et al., 2020) and agreed with a concomitant study of 4,000 *Burkholderia s.l.* genome assemblies (Mullins and Mahenthiralingam, 2021). To formally describe a new bacterial species according to the rules of the ICSP, the type-strain should be deposited in two international culture collections (Garrity et al., 2015). Thus, there is a gap between finding a potentially new species in a genome dataset and formally describing it. After the effective description, the new species name should be validated by the ICSP. All not validly published species should be mentioned within quotation marks, following the List of Prokaryotic names with Standing in Nomenclature (LPSN). In this work, we provided an updated taxonomy of *Burkholderia s.l.* and used genome metrics to evaluate whether recently described novel species could be validly accepted and if closely related species should be considered synonyms. 

Many genome metrics are available for species delineation (Sant’Anna et al., 2019), especially variations of ANI (Palmer et al., 2020). Whole-genome ANI pairwise comparisons are most commonly performed through BLASTn or MUMmer alignment algorithms, named ANIb and ANIm, respectively (Richter and Rosselló-Móra, 2009). While ANIm is advantageous for preliminary analysis of extensive sequence data, ANIb shows more robust results (Richter and Rosselló-Móra, 2009). To follow the previous recommendation of the ICSP to evaluate taxonomic relatedness with two different metrics (Wayne et al., 1987), the use of both ANI and dDDH should be considered since they measure different genome properties especially depending on the chosen dDDH formula (Auch et al., 2010; Li et al., 2015; Volpiano et al., 2021). It is important to note that GGDC provides dDDH values calculated with different formulae (Meier-Kolthoff et al., 2013), dDDH formula 1 (GBDP formula *d*_*0*_ ), formula 2 (GBDP formula *d4*), and dDDH formula 3 (GBDP formula *d*_*6*_ ). Formula 2 is recommended for evaluating incompletely sequenced genomes, which comprise a large proportion of the current sequences available in genomic databases.

**Figure 1 -:**
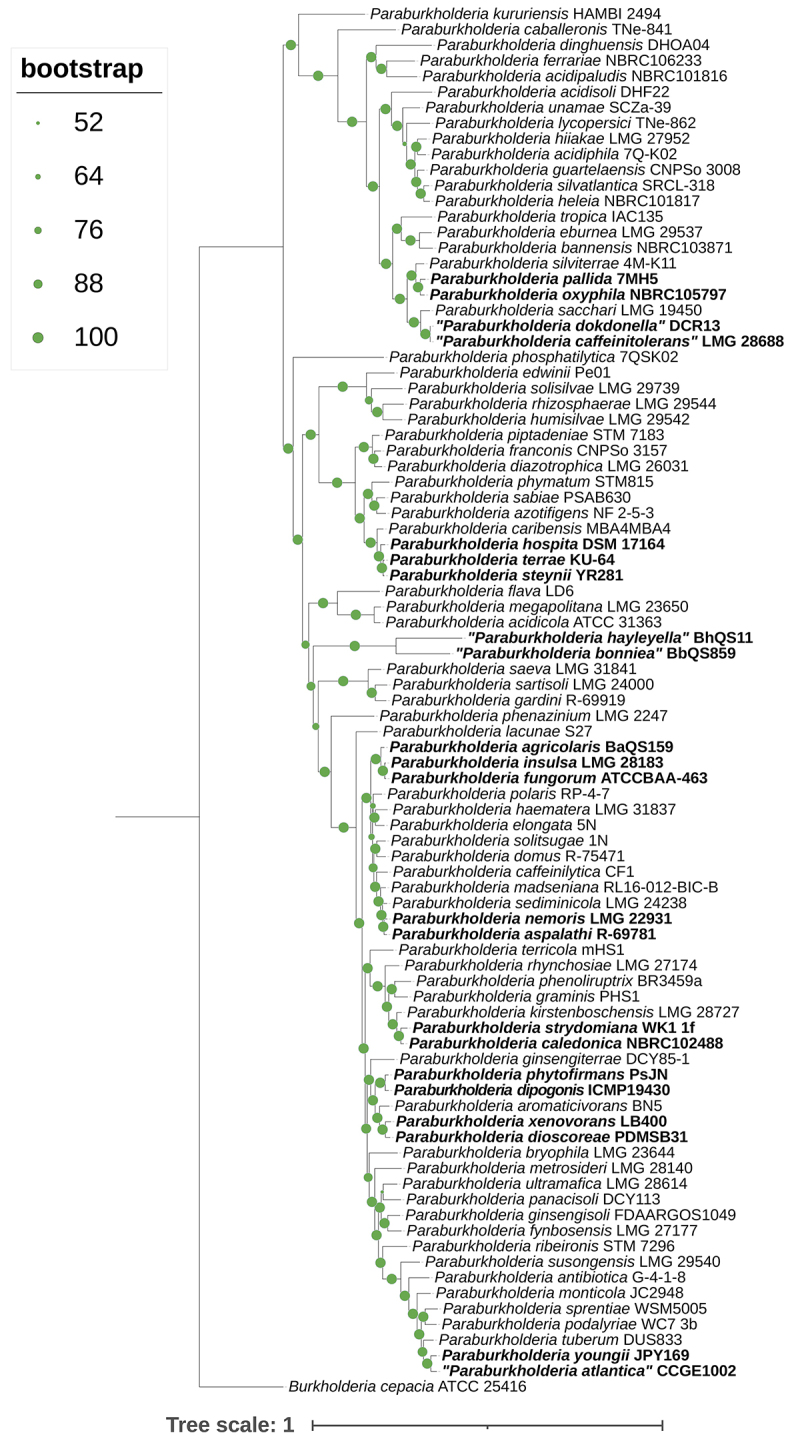
Phylogenetic tree of representative *Paraburkholderia* spp. based on the alignment of 273 orthologous protein sequences recognized by GET-HOMOLOGUES and reconstructed through the maximum likelihood approach of GET-PHYLOMARKERS. Borderline species investigated in this work were highlighted in bold. *Burkholderia cepacia* ATCC 25416^T^ was set as the outgroup. All bootstrap values are shown.

Our previous work agreed with Jin et al. (2020) findings, which indicated that dDDH was more discriminatory for *Burkholderia* species delineation. Both studies showed that some *Burkholderia* type species exhibit ANI values above the 95% threshold while sharing dDDH values below the 70% cutoff for species delineation. Diagnostic differential phenotypic traits corroborate the validity of these strains as distinct species. ANI values above the proposed threshold of 95% were also observed among pairwise comparisons of different type species when Mullins and Mahenthiralingam (2021) investigated 4,000 *Burkholderia s.l.* genome assemblies. These authors recommended the adjustment of the ANI threshold to 96% to discriminate *Paraburkholderia fungorum* from *Paraburkholderia agricolais*; *Paraburkholderia caledonica* from *Paraburkholderia strydomiana*; *Paraburkholderia phytofirmans* from *Paraburkholderia dipogonis*; *Paraburkholderia hospita* from *Paraburkholderia steynii* and *Paraburkholderia terrae,* while the last two could be differentiated using the cutoff value of 97%. Here we show that these species are effectively discriminated by evaluating both ANI and dDDH with the universal threshold values of 95% and 70%, respectively (Table 1). Moreover, phylogenomics also separated these species into distinct clades (Figure 1). Exceptions will be highlighted below.

**Table 1 -:** Pairwise whole genome comparisons performed in this work and taxonomic recommendations for *Burkholderia sensu lato* strains.

Putative type strains	Reference strains	JSpecies		FastANI	dDDH			Proposition
		ANIm	ANIb		formula 1	formula 2	formula 3	
*Paraburkholderia insulsa* LMG 28183	*Paraburkholderia fungorum* LMG 16225	**98.51**	**97.57**	**98.42**	**74.80**	**85.80**	**79.40**	*P. insulsa* is a later heterotypic synonym of *P. fungorum*
*Paraburkholderia agricolaris* BaQS159	*Paraburkholderia fungorum* LMG 16225	**95.83**	**94.06**	**95.55**	56.70	64.60	59.10	Different species
*Paraburkholderia dipogonis* ICMP 19430	*Paraburkholderia phytofirmans* PsJN	**95.95**	94.17	**95.80**	55.20	66.10	57.90	Different species
*Paraburkholderia terrae* DSM 17804	*Paraburkholderia hospita* DSM 17164	**95.42**	93.83	**95.17**	61.70	62.00	63.20	Different species
*Paraburkholderia steynii* HC1.1ba	*Paraburkholderia hospita* DSM 17164	**95.13**	94.2	94.56	54.40	59.70	56.10	Different species
*Paraburkholderia steynii* HC1.1ba	*Paraburkholderia terrae* DSM 17804	**96.78**	**96.0**	**96.52**	60.60	**71.2**	63.90	Different species
*Paraburkholderia strydomiana* WK1.1f	*Paraburkholderia caledonica* LMG19076	**95.86**	94.42	**95.63**	65.90	65.50	67.70	Different species
*Paraburkholderia aspalathi* LMG 27731	*Paraburkholderia nemoris* LMG 31836	**95.73**	94.22	**95.64**	61.8	64.9	63.9	Different species
*Paraburkholderia dioscoreae* PDMSB31	*Paraburkholderia xenovorans* LB400	94.9	92.8	94.70	56.3	58.4	57.6	Different species
*Paraburkholderia pallida* 7MH5	*Paraburkholderia oxyphila* NBRC 105797	**95.65**	93.53	**95.32**	56.8	63.6	59	Different species
*“Paraburkholderia atlantica”* CCGE1002	*Paraburkholderia youngii* JPY169	94.92	93.14	94.57	57.7	59.6	59	“*P. atlantica*” could be validly accepted**
*“Paraburkholderia caffeinitolerans”* LMG 28688	*“Paraburkholderia dokdonensis”* DCR-13	**99.94**	97.82*	**99.77**	50.1	**98.4**	56.9	“*P. caffeinitolerans*” could be validly accepted**; “*P. dokdonensis*” belongs to the same species
*Burkholderia oklahomensis* C6786	*“Burkholderia mayonis” BDU6*	**95**	94.23	**95.08**	68.6	58.5	68.5	*“B. mayonis”* could be validly accepted**
*Burkholderia orbicola* TAtl-371	*Burkholderia cenocepacia* NCTC 13227	**95.12**	93.94	**95.21**	69.60	59.60	69.70	Different species
*“Burkholderia semiarida”* CCRMBC74	*Burkholderia cenocepacia* NCTC 13227	94.38	93.8	94.19	59.5	55.5	59.8	*“B. semiarida”* could be validly accepted**
*“Burkholderia semiarida”* CCRMBC74	*Burkholderia orbicola* TAtl-371	94.35	93.9	94.36	**70.3**	55.3	69.2	
*“Burkholderia semiarida”* CCRMBC74	*“Burkholderia sola”* CCRMBC51	94.36	93.86	94.44	**73.7**	55.9	**72.2**	
*“Burkholderia sola”* CCRMBC51	*Burkholderia cenocepacia* NCTC 13227	94.61	94.04	94.53	62.1	57.1	62.5	*“B. sola”* could be validly accepted**
*“Burkholderia sola”* CCRMBC51	*Burkholderia orbicola* TAtl-371	94.78	94.41	94.83	**70.7**	58.8	**70.5**	
*Burkholderia plantarii* ATCC 43733	*“Burkholderia perseverans”* INN12	**95.47**	94.73	**95.98**	**76.7**	60.6	**76.1**	*“B. perseverans”* could be validly accepted**
*Burkholderia mallei* ATCC 23344	*Burkholderia pseudomallei* ATCC 23343	**99.17**	98.8*	**99.29**	**83.1**	**92.5**	**87.6**	Same species; *B. mallei* exhibits a genome reduction
*“Burkholderia reimsis”* BE51	*Burkholderia cepacia* ATCC 25416	**97.65**	**96.84**	**97.48**	**78.10**	**77.90**	**80.90**	“*B. reimsis*” was misidentified. Strain BE51 belongs to *B. cepacia*
*Caballeronia terrestris* LMG 22937	*Caballeronia humi* LMG 22934	94.99	93.65	94.60	69.60	59.30	69.60	Different species
	*Caballeronia humi* KEMC 7302-068	84.6*	75.63*	80.53	17	22.10	16.9	The genomic assembly GCF_007474635.1 of KEMC 7302-068 should be avoided
*Caballeronia humi* LMG 22934	*Caballeronia humi* KEMC 7302-068	84.6*	75.6*	80.56	17.30	22	17.20	

Bold values are above the cutoff for species delineation; * Alignment fractions below 65%; **Could be validly accepted as a new species once other ICSP requirements are met.

*Burkholderia s.l.* genomes exhibiting ANI borderline values were detected with FastANI followed by ProKlust analysis (Figure S1 and Table S3) and pangenome analyses (Figure S2). By further using ANI and dDDH, our results confirmed i) the presence of a synonym within *Burkholderia s.l.;* ii) reinforced that some *Paraburkholderia* are distinct species that share ANIb values >95%; and iii) recommended the acceptance of new species. Our results and some other recommendations for the taxonomic study of this group are detailed below.

### ANI and dDDH discriminate *Paraburkholderia* spp. 

Some *Paraburkholderia* species share ANI values within the threshold for species circumscription (95-96%), forming identity clusters in the ProKlust analysis (Figure S1, Table S3): *Paraburkholderia insulsa, P. agricolaris*, and *P. fungorum*; *P. dipogonis* and *P. phytofirmans; P. hospita, P. steynii*, and *P. terrae*; *P. strydomiana* and *P. caledonica; Paraburkholderia aspalathi* and *Paraburkholderia nemoris; Paraburkholderia pallida* and *Paraburkholderia oxyphila* (Table 1). The close relationship of these species could also be observed through phylogenomics (Figure 1). However, most of these genome sequences share dDDH values below the 70% threshold. 

In accordance with our results, the authors who described the forest soil isolate *P. nemoris* as a new species (Vanwijnsberghe et al., 2021), observed orthoANI values above the species threshold and dDDH values below the species cutoff when compared to *P. aspalathi* (former *Burkholderia aspalathi*) (Mavengere et al., 2014). Similarly, the forest soil species *P. pallida* and *P. oxyphila* (former *Burkholderia oxyphila*) shared ANI values above 95% and dDDH values below 65%. Noteworthily, the use of ANI metrics alone would not be enough to separate these closely related species. Therefore, we highlight that the combined investigation of ANI and dDDH is useful for discriminating closely related *Paraburkholderia* species.

*Paraburkholderia agricolaris* is a soil-dwelling amoebae symbiont (Brock et al., 2020), while *P. terrae* and *P. hospita* type strains were isolated from soil and have similar genomic features and eco-phenotypes, interacting with soil fungi (Pratama et al., 2020). *Paraburkholderia steynii, P. strydomiana*, and *P. dipogonis* are among the plant symbionts included in the symbiovars sv. africana owing to their capacity to nodulate Papilionoideae legumes from South Africa and New Zealand (Paulitsch et al., 2020b), leading to their classification into the sv. Papilionideae (Bellés-Sancho et al., 2023). The studies that described these strains as new species also mentioned high 16S rRNA gene and ANI identity values (Sheu et al., 2015; Beukes et al., 2019). For instance*, P. steynii* and *P. terrae* type strains and *P. strydomiana* and *P. caledonica* share a similarity of 100% in the 16S rRNA gene sequence (Beukes *et al.*, 2019). Therefore, classifying strains into these species might be problematic without genome metrics. These authors could only differentiate the new species using conventional and digital DDH. Similarly, we showed that *P. strydomiana* and *P. caledonica* shared ANIb and dDDH values below species boundaries (Table 1) and should be considered different species following Wayne et al. (1987) rationale. These authors showed that genomically similar isolates formed monophyletic clades in the phylogenetic reconstructions with the new species, reinforcing them as distinct species. However, dDDH formula 2 among *P. steynii* and *P. terrae* was above the threshold for species circumscription (71.2%; 68.4 to 74.3% of confidence interval). 

### An exception given to *P. steynii* and *P. terrae*

By evaluating the taxonomic status of *P. terrae* and *P. steynii* with care, we observed that they could not be differentiated by combining the evaluation of the universal threshold values for ANI and dDDH. The original work that describes *P. steynii* as a new species provides comparisons with *P. terrae* type strain showing some phenotypic differences, DDH values below 70%, and average dDDH values of 65.2% (Beukes et al., 2019). However, it is well known that phenotypic tests and the conventional DDH methodology are unreliable. Besides that, it is uncommon to consider the average of the three dDDH methodologies for species circumscription. These authors also highlight a remarkable difference among not only *P. steyni* and *P. terrae*, but also among *P. strydomiana* and *P. caledonica*: both *P. strydomiana* and *P. steynii* were able to nodulate the leguminous plant *Hypocalyptus sophoroides*, whereas the closely related type strain *P. caledonica* NBRC 102488^T^ and *P. terrae* NBRC 100964^T^ could not (Beukes *et al.*, 2019). The inability of nodulation of these strains was claimed since the authors could not find the common nodulation loci, *nodABCD*, in their genomes. 

**Figure 2 -:**
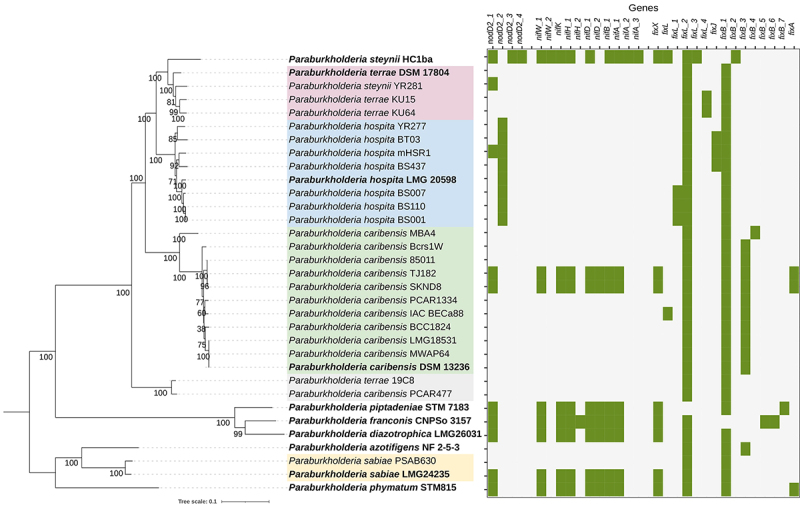
Phylogenetic tree of *Paraburkholderia* strains and heatmap showing the presence (green) of the symbiotic genes *nod, nif,* and *fix* in each genome sequence. The phylogeny was based on the alignment of 88 orthologous protein sequences recognized by GET-HOMOLOGUES and reconstructed through the maximum likelihood approach of GET-PHYLOMARKERS. Type strains are shown in bold. *Paraburkholderia phymatum* STM815^T^ was set as the outgroup. All bootstrap values are shown.

To reinforce this finding, we annotated with Prokka all available genome sequences of *P. steynii*, *P. terrae*, *P. hospita*, *Paraburkholderia caribensis,* and other closely related species and performed pangenome analysis. Figure 2 shows that *P. steynii* HC1ba^T^ harbors a similar profile of *nod* and *nif* genes compared to two strains of *P. caribensis,* and the type strains of *Paraburkholderia piptadeniae, Paraburkholderia franconis, Paraburkholderia diazotrophica,* and *Paraburkholderia sabiae,* all South American mimosoid-nodulating species. *Paraburkholderia hospita* strains harbor a different *nodD* gene. A recent review describes differences in the origin of symbiotic genes of *Paraburkholderia* spp. isolated from nodules of South American and South African legumes (Bellés-Sancho et al., 2023). Regarding our taxonomic focus, we could show that *P. terrae* strains lack *nodD* and *nif* genes. Even though the type strain was characterized as a new nitrogen-fixing (diazotrophic) species by cultivating it in a nitrogen-free medium and amplifying the *nifH* gene through PCR (Yang et al., 2006), we could not confirm this data through genome analysis. The ability to nodulate legumes could be a significant difference among these closely related strains. Thus, these species are kept separated and constitute an exception for our dataset since they could not be differentiated using the universal cutoffs of ANI and dDDH. As mentioned above, an adjusted ANI threshold of 97% was proposed to delineate these species (Mullins and Mahenthiralingam, 2021). 

A previous proposal reclassified these strains as *P. terrae* subspecies *terrae* and *P. terrae* subspecies *steynii* due to some differential phenotypic traits (Madhaiyan et al., 2022). However, we have some concerns about this proposition. The subspecies rules are less clear than the bacterial species circumscription. For instance, there is a proposal to delineate subspecies based on a dDDH threshold of 79% (Meier-Kolthoff et al., 2014), which is far from the value obtained for these strains. Moreover, the annotation file of *P. steynii* HC1ba^T^ has been recently removed from RefSeq, raising concerns regarding genome quality. Of note, the subspecies status is shown as not validated in the LPSN website, while the GTDB (Genome Taxonomy Database) considers them as synonyms. These issues could be clarified once more genomes of *P. steynii* strains are sequenced. 

Considering that the genome of *P. steynii* should contain *nod* and *nif* genes, we suggest reclassifying strain YR281 to *P. terrae* due to the absence of *nif* and some *nodD* genes in its genome. Moreover, YR281 shares higher ANI values with other *P. terrae* strains (above 97%) than with *P. steynii* (96.3%). All suggested reclassifications of non-type strains of this work are shown in supplementary table S2. Two strains, *P. terrae* 19C8 and *P. caribensis* PCAR477, were grouped in clusters based on phylogenomic and ANI analyses (Figures 2 and S3). These strains showed ANIb values below 95% compared to other *Paraburkholderia* type species and shared ANI similarities of 98.7% among them (Figure S3). Therefore, they belong to a new *Paraburkholderia* species. 

### *Paraburkholderia insulsa* as a later heterotypic synonym of *Paraburkholderia fungorum*

Our results indicated that these two species shared an ANIb value of 97.57%, ANIm of 98.51%, and dDDH values >74.8% (Table 1). The whole genome phylogenetic reconstruction also indicates that these species are highly similar, sharing the same most recent ancestor (Figure 1). *Paraburkholderia fungorum* was initially isolated from the white-rot fungus *Phanerochaete chrysosporium*, but was later found in various human and veterinary clinical samples (Coenye et al., 2001). It was described as a new species of *Burkholderia* in 2001 and then moved to the new genus *Paraburkholderia* according to phylogenetic clustering (Dobritsa and Samadpour, 2016). In 2015, Rusch et al. (2015) proposed the new species *P. insulsa* as a unique strain isolated at 30 m distance from an arsenic-rich hydrothermal vent in Papua Nova Guinea. This strain showed high 16S rRNA gene similarity with *P. fungorum* (99.8%), *P. phytofirmans* (98.8%), *P. caledonica* (98.4%), and *Paraburkholderia sediminicola* (98.4%), all previously belonging to *Burkholderia*. A few phenotypic differences were observed among them, including lipid composition, carbohydrate utilization, and enzyme profiles. However, DDH values indicated that *P. insulsa* PNG-April was a different species due to reassociation values below the 70% threshold (35-36.7% with *P. fungorum* DSM 17061^T^, 10.3 and 20.5% with *P. phytofirmans* DSM 17436^T^). Since DDH exhibits low reproducibility, this value may not be reliable. Besides that, minor phenotypic differences could be a result of intraspecies differences. Therefore, according to the genome similarities found here and in previous works (Mullins and Mahenthiralingam, 2021; Bach et al., 2022b), *P. insulsa* (Rusch *et al.*, 2015) should be considered a later heterotypic synonym of *P. fungorum* (Coenye *et al.*, 2001). Similarly, recent work has made the same proposition (Madhaiyan et al., 2022).

###  Phylogenomics and genome metrics indicate that “*Paraburkholderia atlantica*”, “*Paraburkholderia caffeinitolerans*”, “*Paraburkholderia bonniea*”, and “*Paraburkholderia hayleyella*” indeed represent new species 

Our phylogenomic and ANI analyses (Figures 1 and S2) indicated that, at least from a genomic standpoint, the species “*P. atlantica*”, “*P. bonniea*”, “*P. caffeinitolerans*”, and “*P. hayleyella*” represent new species (Gao et al., 2016; Brock et al., 2020; Paulitsch et al., 2020a). Despite displaying near-cutoff ANI values, not only *Paraburkholderia dioscoreae* and *Paraburkholderia xenovorans* but also *Paraburkholderia youngii* and *“P. atlantica”* could be differentiated by dDDH*.* Hence, based on genome metrics, the Brazilian Atlantic Forest species “*P. atlantica*” (Paulitsch *et al.*, 2020a) could be validly accepted as a new species once other ICSP requirements are met. Both “*P. atlantica*” and *P. youngii* were previously classified as *Paraburkholderia tuberum* sv. mimosae and formed clearly separated clusters in MLSA and ANI analyses. Interestingly, these species contain strains able to fix nitrogen and nodule mimosoid legumes of South and Central America, which also led to their classification into the symbiovar sv. atlantica (Mavima et al., 2022). 

“*Paraburkholderia caffeinitolerans*” was isolated from a Chinese tea plantation soil and showed caffeine degrading abilities (Gao et al., 2016). Years later, a Korean strain isolated from the rhizosphere of *Campanula takesimana* was described as the new species “*Paraburkholderia dokdonella*” (Jung et al., 2019). However, genome metrics indicated that “*P. dokdonella*” is a later heterotypic synonym of “*P. caffeinitolerans*”, which could be validly accepted as a new species. This result corroborated previous findings (Mullins and Mahenthiralingam, 2021). Madhaiyan et al. (2022) have recently reinforced the proposal of “*P. dokdonella”* as a new species by correcting its name to “*Paraburkholderia dokdonensis*” and providing culture collection deposit certificates. However, we have some concerns regarding this genome sequence. “*Paraburkholderia dokdonensis*” shows an atypical genome size (4.4 Mbp) compared to other *Paraburkholderia* spp. (7-10 Mbp), which resulted in ANIb alignment fractions of 57%. It remains to be evaluated if this is due to a genome reduction or an anomalous genome assembly. 

In general, bacterial endosymbionts, intracellular pathogens, or obligate pathogens harbor reduced genomes (González-Torres et al., 2019). For instance, *Paraburkholderia agricolaris, “P. bonniea”, and “P. hayleyella*” were isolated from the amoebae *Dictyostelium discoideum* and were found to remain in symbiosis during all host life stages. Both “*P. bonniea”* and “*P. hayleyella”* harbor reduced genome sizes probably related to gene losses commonly associated with an adaptation to the symbiotic lifestyle (Brock et al., 2020). Intriguingly, their G+C content (58.7 and 59.2%) was also lower than other *Burkholderia s.l* (Table S2), except for *Robbsia andropogonis*, whose genome G+C content is 59.1%. ANI values between “*P. bonniea”* and “*P. hayleyella”* and *R. andropogonis* are 77 and 76.7%, respectively. “*Paraburkholderia bonniea*” and “*P. hayleyella*” lack culture collection deposit certificates, and thus are still not validly published. 

Likewise, the fungal endosymbionts *Mycetohabitans* spp. exhibit genome sizes ranging from 3.2 to 3.8 Mbp. Another well-known example of genome reduction within *Burkholderia s.l*. is *B. mallei*, the obligate pathogen that causes glanders in horses, occasionally also infecting humans and other animals (Godoy et al., 2003). Since the proposal of *Burkholderia* as a new genus in 1992, *B. mallei* and *B. pseudomallei* are recognized as a single species (Yabuuchi et al., 1992). This could be observed using ProKlust FastANI clusters, phylogenomics, and additional genome metrics (Figure S4 and Tables 1 and S3). Ideally, these strains should be reclassified as *B. mallei* subspecies *mallei* and *B. mallei* subspecies *pseudomallei*. However, they are historically kept as different species due to the differences in the disease they cause. While *B. mallei* is an obligate pathogen mainly affecting horses, *B. pseudomallei* opportunistically causes melioidosis in humans and other animals (Godoy *et al.*, 2003). *Burkholderia mallei* ATCC 23344^T^ harbors a genome of 5.8 Mbp, whereas the genome of *B. pseudomallei* ATCC 23343^T^ has a size of 7 Mbp.

### Phylogenomics and genome metrics indicate that “*Burkholderia mayonis*”, “*Burkholderia semiarida*” and “*Burkholderia sola*” could be validated as new species 

“*Burkholderia mayonis”* is a soil isolate from tropical northern Australia and was characterized as a new species of the *B. pseudomallei* complex through biochemical and genomic differences (Hall et al., 2022). Even though “*B. mayonis*” shares ANI values of 95% with *Burkholderia oklahomensis*, dDDH discriminated them as separate species (Table 1). “*Burkholderia perseverans*” belongs to the cluster of plant pathogens such as *Bukholderia glumae, Bukholderia gladioli,* and *Bukholderia plantarii* (Figure S4). “*Burkholderia perseverans*” was isolated from leaf litter of Brazilian’s Restinga ecosystem and exhibits antifungal properties. It was differentiated from *B. plantarii* type strain through ANI, dDDH, and biochemical tests, including lack of growth in TSA medium containing 3% NaCl, citrate assimilation, and β-galactosidase activity (Andrade et al., 2021). These authors also characterized another two “*B. perseverans*” isolates that formed a separated cluster in the phylogenetic tree. 

Here we show that differentiating “*B. perseverans*” from *B. plantarii* using genome metrics requires attention. Despite the widely used ANIb and dDDH formula 2 being below the species circumscription cutoffs, this is not the case for ANIm and the other two dDDH formulae (Table 1). If the criterium of using two metrics that measure different genomic properties is considered (Wayne et al., 1987), we should consider ANI and dDDH formulae 1 or 3. Formula 2 is similar to ANI and is especially recommended when comparing draft genomes, which is not true for these sequences (Table S2). Therefore, using this criterium, we should consider *B. plantarii* and “*B. perseverans*” the same species. However, we agree with the proposal of the new species (Table 1) due to the following data: i) both genomes are complete and result in ANIb values below 95%. ANIb is more robust and the current preferential ANI methodology; ii) dDDH formula 2 is below 70% and is the developer’s recommended formula; iii) other “*B. perseverans*” isolates formed a separated clade in the phylogenetic tree; and iv) they exhibit diagnostic phenotypic traits (Andrade et al., 2021). This case highlights the eminent necessity for establishing more precise and detailed criteria for species delineation using genome metrics by the ICSP.

In 1997, multiple genomovars (I-V) were recognized within *B. cepacia* isolated from cystic fibrosis patients by whole-cell protein electrophoresis, DDH, and other phenotypic traits (Vandamme et al., 1997). *Burkholderia cenocepacia* was later proposed for “*B. cepacia* genomovar III”, encompassing the r*ecA* gene lineages IIIA, IIIB, IIIC, and IIID (Vandamme *et al.*, 2003). These authors have already described DDH values of 58-83% among strains of lineages IIIA and IIIB. In our previous pangenome analysis (Bach et al., 2022b), these two clusters were also clearly separated by ANI, dDDH and phylogenomics. The cluster containing the type strain was called *B. cenocepacia* BCC08, while the other cluster was called *Burkholderia* sp*.* BCC05, corresponding to *recA* lineages IIIA and IIIB, respectively. ANI values between the strains from these groups were above 97.7%, while values among groups were in the twilight zone (95.4-95.7%). Likewise, dDDH values of type strain *B. cenocepacia* NCTC13227^T^ were above 89.9% within-cluster BCC08 (IIIA) and below 60.4% compared to strains belonging to cluster BCC05 (IIIB). Therefore, we suggested that BCC05 represents a new species. 

**Figure 3 -:**
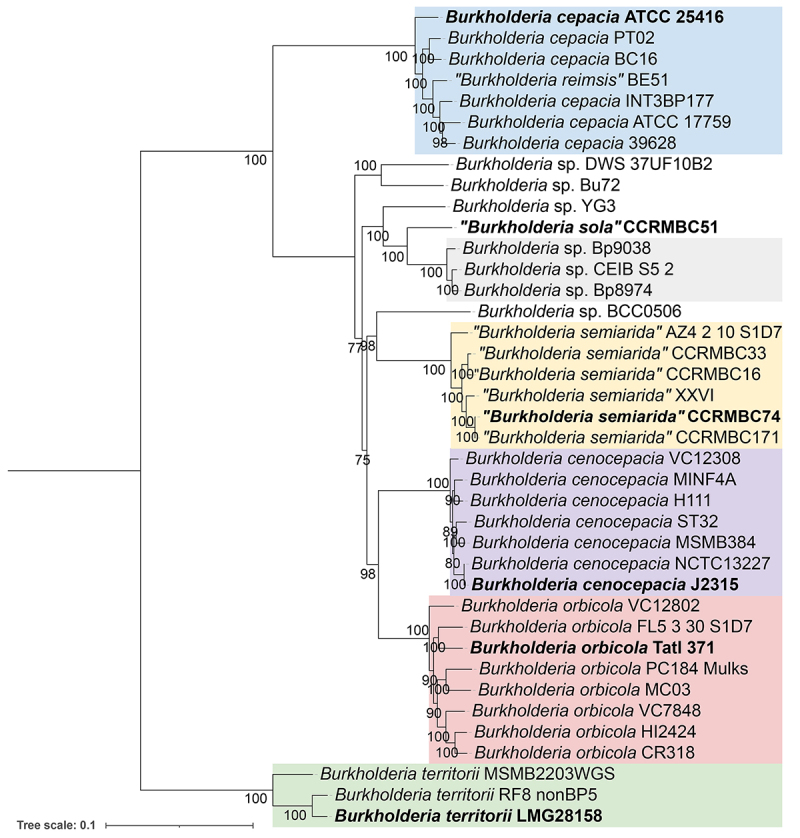
Phylogenetic tree of *Burkholderia* strains based on the alignment of 695 orthologous marker sequences recognized by GET-HOMOLOGUES and reconstructed through the maximum likelihood approach of GET-PHYLOMARKERS. Type strains are shown in bold. *Burkholderia territorii* strains were set as the outgroup. All bootstrap values are shown.

In addition to recognizing these two clusters through ANIb and dDDH pairwise comparisons, Wallner et al. (2019) observed differences in the genome size, G+C content and protein coding sequence regions among them. More importantly, these authors evaluated gene content and observed that genomes from cluster BCC05 (IIIB) lack virulence traits present in BCC08 (IIIA) genomes, which was composed of clinical strains. They proposed the new species *“Burkholderia servocepacia”* to accommodate strains isolated from diverse sources (e.g., hospital, agricultural soil) that formed cluster IIIB. In 2022, Morales-Ruíz et al. (2022) performed genome metrics, phenotypic, and chemotaxonomic characterizations to accomplish the current mandatory rules for bacterial species descriptions and renamed “*B. servocepacia”* to *Burkholderia orbicola,* which is currently a validly accepted name. Here we extended our previous analyses to include the strain proposed as type, TAtl371. This strain clustered within BCC05 in ANI, dDDH, and phylogenomic analyses (Table 1, Figures 3 and S5). 

Similarly, we have previously shown that the Bcc strains AZ4-2-10-S1D7 and XXVI belonged to new species, which we had called BCC03 (Bach et al., 2022b). More recently, two novel Bcc species were described isolated from the semi-arid north-east Brazilian region causing onion sour skin: “*Burkholderia sola*” and “*Burkholderia semiarida*” (Velez et al., 2023). The latter groups with the BCC03 strains in ANI and phylogenetic analyses (Figures 3 and S5). Here we show that, from the genomic standpoint, both species could be validly accepted (Table 1). Besides that, some other new species are still to be described in the vicinity of *B. cenocepacia* (Table S2). A better definition and characterization of strains belonging to this group is critical since *B. cenocepacia* infections of cystic fibrosis patients exhibit poor outcomes (Pope et al., 2010).

### Other recommendations

Genome metrics are revolutionizing bacterial taxonomy since the developed tools are easy to use, the analyses are portable, and the definition of clear thresholds makes the results more reliable. However, there are some concerns regarding the quality of genome sequences and the use of validly published type strains in the comparisons. To propose a new species, a representative strain is chosen as the type strain, which serves as a reference in subsequent taxonomical studies (Chun et al., 2018). Much relevant information about microbial species, such as the type strains, can be found on the LPSN website (https://www.bacterio.net/).

Noteworthily, one should always verify the quality of the compared genomes. Likewise, evaluating the alignment fraction or the percentage of aligned nucleotides is an essential quality check in genomic comparison analysis for taxonomic purposes (Li *et al.*, 2015). In this study, all ANIb and ANIm results showed more than 65% of aligned nucleotides, except for “*B. dokdonensis*”, “*B. mallei*”, and the anomalous assembly of *C. humi* KEMC 7302-068^T^ (Table 1). Noteworthily, genomic databases are improving the curation process and removing anomalous assemblies, but there are still some errors that could mislead taxonomic analyses. The following are some examples within *Burkholderia s.l.*

### “*Burkholderia reimsis*” BE51 belongs to *B. cepacia*

Strain BE51 was suggested as a new species in a genome announcement study without providing further evidence (Esmaeel et al., 2018). This genome is currently assigned as a “representative genome” in the RefSeq database. Our phylogenomic studies indicated that this strain was misidentified (Figure 3). Indeed, genome metrics ANI and dDDH showed that “*Burkholderia reimsis*” BE51 is a *B. cepacia* strain (Table 1 and S3). Therefore, the species “*Burkholderia reimsis*” should not be validly published.

### Incongruencies between “*Burkholderia paludis*” MSh1 ^T^ **genome sequences**

Although our phylogenomic studies did not indicate problems with the genome sequence of “*B. paludis*” MSh1^T^, Peeters et al. (2020) recently called attention to incongruencies with the sequence type gene markers obtained from the deposited genome of “*B. paludis*” MSh1^T^, the resequenced genome of LMG 30113^T^, and the information provided in the original description paper (Ong et al., 2016). Therefore, one should interpret genomic data from this species cautiously, and the name should only be validly published if these incongruencies are solved.

### The genome assembly of *Caballeronia humi* KEMC 7302-068 should be avoided 

*Caballeronia terrestris* LMG 22937^T^ and *Caballeronia humi* LMG 22934^T^ share high ANIb (93.65%) and dDDH values (69.60%), yet below species boundaries (Table 1), confirming that they are separate species. The NCBI RefSeq database correctly indicates the genome assembly GCF_001544475.1 of *C. humi* LMG 22934^T^ as the representative genome. However, another deposit of *C. humi* KEMC 7302-068^T^, GCF_007474635.1, might be used preferentially by researchers seeking genomes with higher completeness, which is the case of the latter (50% instead of 25% of NCBI’s classification). Furthermore, here we showed that the assembly GCF_007474635.1 shows discrepant ANI and dDDH when compared to GCF_001544475.1 (Table 1), both putatively sequenced from the type strain. Therefore, the genome assembly GCF_007474635.1 should be avoided in taxonomic investigations. This assembly failed the NCBI database taxonomy check and has been recently removed from RefSeq.

## Final remarks

Many studies have revisited the taxonomy of *Burkholderiaceae* using genome-based tools (Dobritsa and Samadpour, 2016; Estrada-de Los Santos et al., 2018; Wallner et al., 2019; Jin et al., 2020; Mullins and Mahenthiralingam, 2021; Bach et al., 2022b). These works have given valuable contributions to understanding the group since the reliable identification of a strain is an important step in exploring biotechnological potentials, being aware of biosafety risks, and choosing the most appropriate clinical protocols. However, we would like to highlight that other phenotypic investigations, beyond genomic analysis, should be performed to confirm the pathogenicity or host specificity of a strain. Furthermore, synonyms and putative new species within this group have already been recognized (Jin *et al.*, 2020; Mullins and Mahenthiralingam, 2021; Bach *et al.*, 2022b). Here we performed additional analyses to corroborate previous results, evaluated the presence of synonyms, and suggested some recommendations for the taxonomic study of this bacterial group. 

ANI results varied among tools due to the implementation of different algorithms or the adoption of slight modifications in the formulae (Table 1). However, these differences should not be a problem since there is a recommendation to evaluate at least two different metrics to delineate species (Wayne et al., 1987). Here we highlighted the importance of using ANI and dDDH as a more discriminatory pipeline for *Burkholderia s.l.* strains that present borderline values in whole-genome comparisons. Thus, we recommend using a combination of the universal thresholds of 95 and 70% for ANI and dDDH calculations, respectively, to delineate species of this group reliably. In some cases, it was also necessary to evaluate phylogeny and the description of differential phenotypic traits that corroborate genomic differences. A previous comprehensive work has proposed using multiple ANI values to discriminate some *Burkholderia s.l.* species unequivocally (Mullins and Mahenthiralingam, 2021). This procedure enables the screening of large datasets once the dDDH tool is limited to a few comparisons per time, hindering its extensive adoption in genome-based taxonomic projects. However, following a universal threshold should be preferential to standardize communication among different areas. Considering these pragmatic criteria for the evaluation of genome metrics, here we revised the current *Burkholderia s.l.* taxonomy and reclassified *P. insulsa* as a later heterotypic synonym of *P. fungorum,* corroborated that closely related strains belong to different species, recommended the validation of species names, and showed incongruencies in names and genome assemblies.

## Supplementary material

The following online material is available for this article:

Figure S1 - Genomic clusters detected using pairwise ANI values from 824 *Burkholderiales* genomes.

Figure S2 - Heatmap of ANI values obtained from pairwise comparisons of genomes of representative *Paraburkholderia* spp.

Figure S3 - Heatmap of ANIb values obtained by pyANI comparison of genomes of selected *Paraburkholderia* strains.

Figure S4 - Phylogenetic tree of *Burkholderia* strains based on the alignment of 467 orthologous protein sequences recognized by GET-HOMOLOGUES and reconstructed through the maximum likelihood approach of GET-PHYLOMARKERS.

Figure S5 - Heatmap of ANI values obtained from pairwise comparisons of genomes of Burkholderia strains.

Table S1 - Sequence data from the genomes of *Burkholderiales* type strains analysed in this work using ProKlust.

Table S2 - List of *Burkholderia s.l.* genomes used in this work together with quality features and reclassifications.

Table S3 - Clusters of identity/similarity formed by the *Burkholderia sensu lato* genomes analysed in this work using FastANI coupled with ProKlust.
