# Supplementary material for: Genome-based taxonomy of Burkholderia sensu lato: Distinguishing closely related species
Source: Genet Mol Biol. 2023 Nov 3;46(3 Suppl 1):e20230122. doi: 10.1590/1678-4685-GMB-2023-0122 (PMC10629849; doi:10.1590/1678-4685-GMB-2023-0122)
Supplement: Figure S2 - [file 1415-4757-GMB-46-3-s1-e20230122-s2.pdf]

Genetics and Molecular Biology
https://doi.org/10.1590/1678-4685-GMB-2023-0122

Supplementary Material to "Genome-based taxonomy of *Burkholderia sensu lato*: distinguishing closely related species"

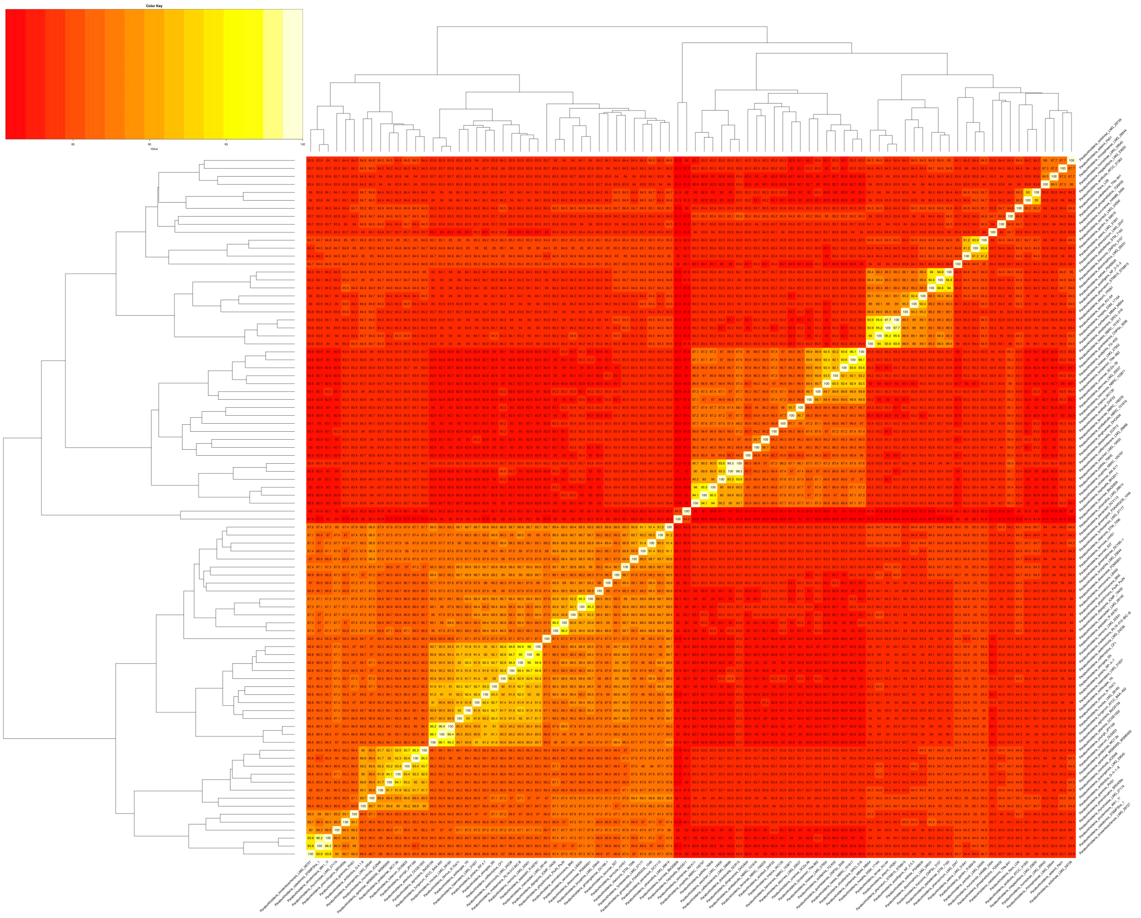

Figure S2 - Heatmap of ANI values obtained from pairwise comparisons of genomes of representative *Paraburkholderia* spp.
