# Supplementary material for: Genome-based taxonomy of Burkholderia sensu lato: Distinguishing closely related species
Source: Genet Mol Biol. 2023 Nov 3;46(3 Suppl 1):e20230122. doi: 10.1590/1678-4685-GMB-2023-0122 (PMC10629849; doi:10.1590/1678-4685-GMB-2023-0122)
Supplement: Figure S4 - [file 1415-4757-GMB-46-3-s1-e20230122-s4.pdf]

Genetics and Molecular Biology
https://doi.org/10.1590/1678-4685-GMB-2023-0122

Supplementary Material to "Genome-based taxonomy of *Burkholderia sensu lato*: distinguishing closely related species"

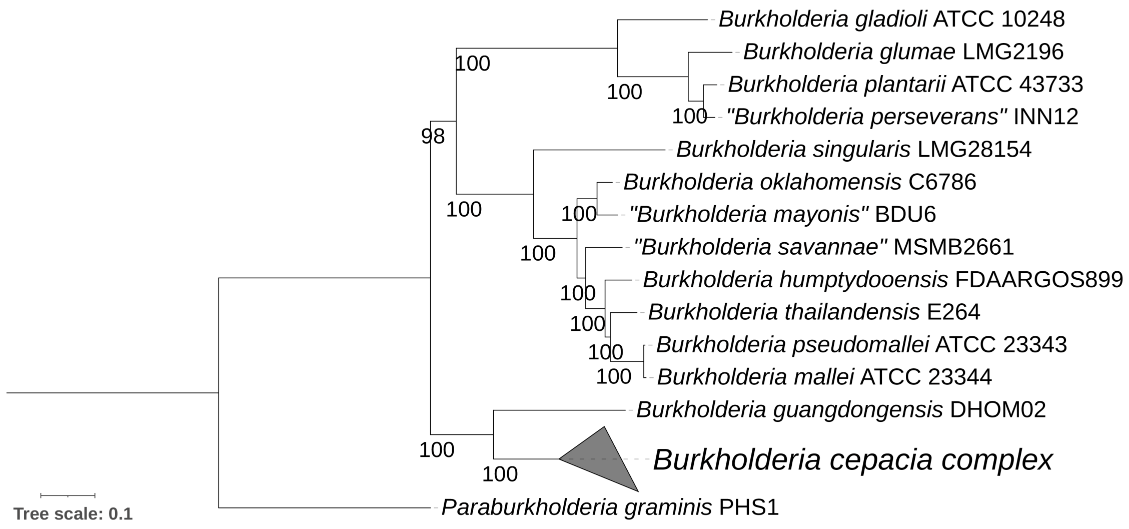

Figure S4 - Phylogenetic tree of *Burkholderia* strains based on the alignment of 467 orthologous protein sequences recognized by GET-HOMOLOGUES and reconstructed through the maximum likelihood approach of GET-PHYLOMARKERS. *Paraburkholderia graminis* PHS1^{T} was set as the outgroup. All bootstrap values are shown.
