# Supplementary material for: Genome-based taxonomy of Burkholderia sensu lato: Distinguishing closely related species
Source: Genet Mol Biol. 2023 Nov 3;46(3 Suppl 1):e20230122. doi: 10.1590/1678-4685-GMB-2023-0122 (PMC10629849; doi:10.1590/1678-4685-GMB-2023-0122)
Supplement: Table S1 - [file 1415-4757-GMB-46-3-s1-e20230122-s6.pdf]

Genetics and Molecular Biology
https://doi.org/10.1590/1678-4685-GMB-2023-0122

Supplementary Material to "Genome-based taxonomy of *Burkholderia sensu lato*: distinguishing closely related species"

Table S1 - Sequence data from the genomes of Burkholderiales type strains analysed in this work using ProKlust.

Assembly accession	Representative genome	Organism name	Type strain name	Assembly level
GCF_000740035.1	na	*Comamonas testosteroni*	NBRC 14951	Contig
GCF_900461225.1	na	*Comamonas testosteroni*	NCTC10698	Contig
GCF_001528845.1	na	*Burkholderia cepacia*	NBRC 14074	Contig
GCF_900446175.1	na	*Burkholderia cepacia*	NCTC10743	Contig
GCF_004342485.1	x	*Pelomonas saccharophila*	DSM 654	Scaffold
GCF_015910705.1	na	*Ralstonia solanacearum*	NCPPB 325	Contig
GCF_000743455.1	na	*Ralstonia pickettii*	ATCC 27511	Scaffold
GCF_016466415.2	na	*Ralstonia pickettii*	K-288	Complete Genome
GCF_020341455.1	na	*Ralstonia pickettii*	FDAARGOS_1535	Chromosome
GCF_900455835.1	na	*Ralstonia pickettii*	NCTC11149	Contig
GCF_002028405.1	x	*Thiomonas intermedia*	ATCC 15466	Complete Genome
GCF_001040945.1	na	*Herbaspirillum seropedicae*	Z67	Complete Genome
GCF_013180375.1	na	*Herbaspirillum seropedicae*	Z67	Contig
GCF_023913775.1	na	*Acidovorax facilis*	DSM 649	Scaffold
GCF_900446245.1	na	*Burkholderia mallei*	NCTC12938	Contig
GCF_002017865.1	x	*Rhodoferax fermentans*	JCM 7819	Contig
GCF_016583655.1	na	*Rhodoferax fermentans*	DSM 10138	Scaffold
GCF_004340905.1	x	*Rubrivivax gelatinosus*	DSM 1709	Scaffold

Assembly accession	Representative genome	Organism name	Type strain name	Assembly level
GCF_016583525.1	na	*Rubrivivax gelatinosus*	DSM 1709	Contig
GCF_000970345.1	na	*Robbsia andropogonis*	ICMP2807	Contig
GCF_902833845.1	x	*Robbsia andropogonis*	LMG 2129	Scaffold
GCF_002879875.1	na	*Trinickia caryophylli*	Ballard 720	Contig
GCF_003350265.1	na	*Trinickia caryophylli*	HAMBI_2159	Scaffold
GCF_007097545.1	x	*Trinickia caryophylli*	DSM 50341	Contig
GCF_902833925.1	na	*Trinickia caryophylli*	LMG 2155	Scaffold
GCF_000959725.1	na	*Burkholderia gladioli*	ATCC 10248	Complete Genome
GCF_900446225.1	na	*Burkholderia gladioli*	NCTC12378	Contig
GCF_001182285.1	na	*Burkholderia pseudomallei*	ATCC 23343	Scaffold
GCF_006538545.1	na	*Burkholderia pseudomallei*	WRAIR 286	Contig
GCF_004342585.1	x	*Paucimonas lemoignei*	DSM 7445	Scaffold
GCF_001758635.1	na	*Janthinobacterium lividum*	H-24	Contig
GCF_020858175.1	na	*Janthinobacterium lividum*	ATCC 12473	Scaffold
GCF_900451145.1	na	*Janthinobacterium lividum*	NCTC9796	Contig
GCF_013267395.1	na	*Achromobacter denitrificans*	FDAARGOS_786	Complete Genome
GCF_900444675.1	na	*Achromobacter denitrificans*	NCTC8582	Contig
GCF_902859715.1	na	*Achromobacter denitrificans*	LMG 1231	Contig
GCF_900461435.1	na	*Comamonas terrigena*	NCTC1937	Contig
GCF_900112675.1	x	*Acidovorax konjaci*	DSM 7481	Scaffold
GCF_900156335.1	na	*Sphaerotilus natans*	ATCC 13338	Scaffold
GCF_020735985.1	na	*Bordetella holmesii*	FDAARGOS_1539	Complete Genome
GCF_900445775.1	na	*Bordetella holmesii*	NCTC12912	Contig
GCF_008801845.1	na	*Ideonella dechloratans*	CCUG 30977	Contig
GCF_021049305.1	x	*Ideonella dechloratans*	CCUG 30977	Complete Genome
GCF_012584515.1	x	*Polaromonas vacuolata*	KCTC 22033	Complete Genome
GCF_003315195.1	na	*Sutterella wadsworthensis*	DSM 14016	Scaffold

Assembly accession	Representative genome	Organism name	Type strain name	Assembly level
GCF_016724805.1	x	*Sutterella wadsworthensis*	FDAARGOS_1159	Complete Genome
GCF_001411805.1	x	*Burkholderia plantarii*	ATCC 43733	Complete Genome
GCF_902832905.1	na	*Burkholderia plantarii*	LMG 9035	Scaffold
GCF_900637555.1	x	*Lautropia mirabilis*	NCTC12852	Complete Genome
GCF_007993815.1	x	*Acidovorax delafieldii*	DSM 64	Scaffold
GCF_004216565.1	x	*Leptothrix mobilis*	DSM 10617	Scaffold
GCF_003217575.1	x	*Xylophilus ampelinus*	CECT 7646	Scaffold
GCF_000698595.1	na	*Caballeronia glathei*	DSM 50014	Contig
GCF_000751215.2	x	*Caballeronia glathei*	LMG 14190	Contig
GCF_900100735.1	x	*Paraburkholderia phenazinium*	LMG 2247	Scaffold
GCF_001028665.1	x	*Burkholderia pyrrocinia*	DSM 10685	Complete Genome
GCF_902832895.1	na	*Burkholderia pyrrocinia*	LMG 14191	Scaffold
GCF_020099355.1	na	*Burkholderia vietnamiensis*	FDAARGOS_1488	Complete Genome
GCF_902830295.1	na	*Burkholderia vietnamiensis*	LMG 10929	Scaffold
GCF_000723405.1	na	*Hydrogenophaga intermedia*	S1	Contig
GCF_005938115.1	na	*Hydrogenophaga intermedia*	DSM 5680	Contig
GCF_008801925.2	x	*Cupriavidus basilensis*	DSM 11853	Complete Genome
GCF_014635845.1	x	*Oxalicibacterium solurbis*	CCM 7664	Contig
GCF_001447195.1	x	*Aquabacterium parvum*	B6	Contig
GCF_004362855.1	x	*Aquabacterium commune*	DSM 11901	Scaffold
GCF_902859685.1	na	*Achromobacter piechaudii*	LMG 1873	Contig
GCF_902859695.1	na	*Achromobacter ruhlandii*	LMG 1866	Contig
GCF_001449005.1	na	*Paraburkholderia caribensis*	MWAP64	Complete Genome
GCF_002902945.1	na	*Paraburkholderia caribensis*	DSM 13236	Complete Genome
GCF_902833515.1	na	*Paraburkholderia caribensis*	LMG 18531	Scaffold
GCF_008801975.1	na	*Castellaniella defragrans*	CCUG 39790	Contig
GCF_014203015.1	na	*Castellaniella defragrans*	DSM 12141	Scaffold

Assembly accession	Representative genome	Organism name	Type strain name	Assembly level
GCF_003387155.1	na	*Roseateles depolymerans*	DSM 11813	Scaffold
GCF_003269065.1	x	*Acidovorax anthurii*	CFPB 3232	Scaffold
GCF_003719195.1	na	*Herbaspirillum rubrisubalbicans*	DSM 11543	Complete Genome
GCF_016127415.1	na	*Delftia acidovorans*	FDAARGOS_997	Complete Genome
GCF_900104515.1	na	*Acidovorax cattleyae*	DSM 17101	Scaffold
GCF_900100305.1	na	*Acidovorax citrulli*	DSM 17060	Scaffold
GCF_006716905.1	x	*Acidovorax temperans*	DSM 7270	Contig
GCF_003568725.1	x	*Simplicispira metamorpha*	NBRC 13960	Scaffold
GCF_004341365.1	na	*Simplicispira metamorpha*	DSM 1837	Scaffold
GCF_001955735.1	x	*Rhodoferax antarcticus*	DSMZ24876	Complete Genome
GCF_008801915.1	na	*Cupriavidus gilardii*	CCUG 38401	Contig
GCF_013004615.1	na	*Cupriavidus gilardii*	ATCC 700815	Contig
GCF_008801835.1	na	*Cupriavidus pauculus*	CCUG 12507	Contig
GCF_019931045.1	na	*Cupriavidus pauculus*	FDAARGOS_1472	Complete Genome
GCF_900107685.1	x	*Paraburkholderia sartisoli*	LMG 24000	Scaffold
GCF_001457475.1	na	*Achromobacter xylosoxidans*	NCTC10807	Complete Genome
GCF_013343135.1	na	*Achromobacter xylosoxidans*	FDAARGOS_789	Complete Genome
GCF_022870085.1	na	*Achromobacter xylosoxidans*	PartM-Axylosoxidans-RM8376	Complete Genome
GCF_002980625.1	x	*Malikia spinosa*	83	Contig
GCF_900446205.1	na	*Burkholderia multivorans*	NCTC13007	Contig
GCF_016028735.1	na	*Oligella ureolytica*	FDAARGOS_872	Complete Genome
GCF_900454285.1	na	*Oligella ureolytica*	NCTC11997	Contig
GCF_900454345.1	na	*Oligella urethralis*	NCTC12964	Contig
GCF_002071575.1	na	*Paraburkholderia tropica*	Ppe8	Scaffold
GCF_900109265.1	na	*Paraburkholderia tropica*	LMG 22274	Scaffold
GCF_902833865.1	na	*Paraburkholderia tropica*	LMG 22274	Scaffold
GCF_001049515.1	na	*Pandoraea apista*	LMG16407	Contig

Assembly accession	Representative genome	Organism name	Type strain name	Assembly level
GCF_001465595.2	x	*Pandoraea apista*	DSM 16535	Complete Genome
GCF_001465545.3	x	*Pandoraea norimbergensis*	DSM 11628	Complete Genome
GCF_000767615.3	na	*Pandoraea pnomenusa*	DSM 16536	Complete Genome
GCF_900454355.1	na	*Pandoraea pnomenusa*	NCTC13160	Contig
GCF_000815105.2	x	*Pandoraea pulmonicola*	DSM 16583	Complete Genome
GCF_900454575.1	na	*Pandoraea pulmonicola*	NCTC13159	Contig
GCF_000814845.2	na	*Pandoraea sputorum*	DSM 21091	Complete Genome
GCF_900187205.1	x	*Pandoraea sputorum*	NCTC13161	Complete Genome
GCF_000067205.1	x	*Bordetella petrii*	DSM 12804	Complete Genome
GCF_001742165.1	na	*Burkholderia stabilis*	ATCC BAA-67	Complete Genome
GCF_900446215.1	na	*Burkholderia cenocepacia*	NCTC13227	Contig
GCF_016894385.1	na	*Cupriavidus oxalaticus*	Ox1	Complete Genome
GCF_902499185.1	na	*Burkholderia ubonensis*		Contig
GCF_902833085.1	na	*Burkholderia ubonensis*	LMG 20358	Scaffold
GCF_900637615.1	na	*Bordetella hinzii*	NCTC13199	Complete Genome
GCF_905397375.1	na	*Ralstonia mannitolilytica*	LMG 6866	Contig
GCF_013133775.1	x	*Pelistega europaea*	LMG10982	Contig
GCF_004342625.1	x	*Tepidimonas ignava*	DSM 12034	Contig
GCF_007556615.1	na	*Tepidimonas ignava*	SPS-1037	Contig
GCF_900078335.1	na	*Bordetella trematum*	NCTC12995	Scaffold
GCF_900445945.1	x	*Bordetella trematum*	NCTC12995	Contig
GCF_902833635.1	na	*Paraburkholderia caledonica*	LMG 19076	Scaffold
GCF_000961515.1	x	*Paraburkholderia fungorum*	ATCC BAA-463	Complete Genome
GCF_902833645.1	na	*Paraburkholderia fungorum*	LMG 16225	Scaffold
GCF_905397395.1	na	*Cupriavidus campinensis*	LMG 19282	Contig
GCF_004216695.1	x	*Pigmentiphaga kullae*	K24	Contig
GCF_019930785.1	na	*Burkholderia dolosa*	FDAARGOS_1463	Complete Genome

Assembly accession	Representative genome	Organism name	Type strain name	Assembly level
GCF_902499135.1	na	*Burkholderia dolosa*	LMG 18943	Contig
GCF_001584145.1	x	*Collimonas fungivorans*	Ter6	Complete Genome
GCF_000785435.2	x	*Paraburkholderia sacchari*	LMG 19450	Contig
GCF_902833715.1	na	*Paraburkholderia sacchari*	LMG 19450	Scaffold
GCF_021026195.1	x	*Comamonas koreensis*	KCTC 12005	Scaffold
GCF_002214645.1	x	*Diaphorobacter nitroreducens*	SL-205	Complete Genome
GCF_900142195.1	na	*Paraburkholderia terricola*	LMG 20594	Scaffold
GCF_902833815.1	na	*Paraburkholderia terricola*	LMG 20594	Scaffold
GCF_002902965.1	x	*Paraburkholderia hospita*	DSM 17164	Complete Genome
GCF_900108355.1	na	*Paraburkholderia hospita*	LMG 20598	Scaffold
GCF_902833685.1	na	*Paraburkholderia hospita*	LMG 20598	Scaffold
GCF_014635065.1	x	*Oxalicibacterium flavum*	CCM 7086	Contig
GCF_016836725.1	na	*Burkholderia anthina*	DSM 16086	Contig
GCF_902498995.1	x	*Burkholderia anthina*		Contig
GCF_902830395.1	na	*Burkholderia anthina*	LMG 20980	Contig
GCF_001189915.1	x	*Herbaspirillum autotrophicum*	IAM 14942	Contig
GCF_900111115.1	x	*Giesbergeria anulus*	ATCC 35958	Scaffold
GCF_002837135.1	x	*Macromonas bipunctata*	DSM 12705	Scaffold
GCF_900102625.1	x	*Acidovorax valerianellae*	DSM 16619	Scaffold
GCF_008801405.1	na	*Ralstonia insidiosa*	CCUG 46789	Contig
GCF_914271545.1	x	*Cupriavidus respiraculi*	LMG 21510	Contig
GCF_004682015.1	x	*Ramlibacter henchirensis*	DSM 14656	Contig
GCF_004216755.1	x	*Kerstersia gyiorum*	DSM 16618	Scaffold
GCF_008801725.1	na	*Kerstersia gyiorum*	CCUG 47000	Contig
GCF_001189955.1	na	*Herbaspirillum chlorophenolicum*	CPW301	Contig
GCF_002933415.1	na	*Schlegelella thermodepolymerans*	DSM 15344	Contig
GCF_003349825.1	na	*Schlegelella thermodepolymerans*	DSM 15344	Scaffold

Assembly accession	Representative genome	Organism name	Type strain name	Assembly level
GCF_004342465.1	na	*Schlegelella thermodepolymerans*	DSM 15264	Scaffold
GCF_015476235.1	x	*Schlegelella thermodepolymerans*	DSM 15344	Complete Genome
GCF_002812705.1	na	*Achromobacter spanius*	DSM 23806	Complete Genome
GCF_902859605.1	na	*Achromobacter spanius*	LMG 5911	Contig
GCF_001971645.1	na	*Achromobacter insolitus*	DSM 23807	Complete Genome
GCF_002209555.1	na	*Achromobacter insolitus*	LMG 6003	Contig
GCF_024168865.1	na	*Achromobacter insolitus*	NCTC 13520	Contig
GCF_900637265.1	na	*Achromobacter insolitus*	NCTC13520	Complete Genome
GCF_902859615.1	na	*Achromobacter insolitus*	LMG 6003	Contig
GCF_905397435.1	x	*Cupriavidus numazuensis*	LMG 26411	Contig
GCF_008801935.1	na	*Comamonas kerstersii*	CCUG 15333	Contig
GCF_000011705.1	na	*Burkholderia mallei ATCC 23344*	ATCC 23344	Complete Genome
GCF_007556585.1	x	*Tepidimonas aquatica*	CLN-1	Contig
GCF_902833655.1	na	*Paraburkholderia phenoliruptrix*	LMG 22037	Contig
GCF_902859825.1	na	*Paraburkholderia phenoliruptrix*	LMG 22037	Contig
GCF_002980595.1	x	*Malikia granosa*	P1	Contig
GCF_002097715.1	x	*Burkholderia ubonensis subsp. mesacidophila*	ATCC 31433	Complete Genome
GCF_002362295.1	na	*Burkholderia ubonensis subsp. mesacidophila*	ATCC 31433	Contig
GCF_000196015.1	na	*Cupriavidus metallidurans CH34*	CH34	Complete Genome
GCF_000013645.1	x	*Paraburkholderia xenovorans LB400*	LB400	Complete Genome
GCF_000756045.1	na	*Paraburkholderia xenovorans LB400*	LB400	Complete Genome
GCF_004217095.1	x	*Advenella incenata*	DSM 23814	Scaffold
GCF_004362525.1	x	*Paucibacter toxinivorans*	DSM 16998	Scaffold
GCF_000012365.1	na	*Burkholderia thailandensis E264*	E264; ATCC 700388	Complete Genome
GCF_000152285.1	na	*Burkholderia thailandensis E264*	ATCC 700388	Chromosome
GCF_003568605.1	na	*Burkholderia thailandensis E264*	E264	Complete Genome

Assembly accession	Representative genome	Organism name	Type strain name	Assembly level
GCF_904848645.1	na	*Paraburkholderia sabiae*	LMG 24235	Contig
GCF_001584165.1	na	*Collimonas arenae*	Ter10	Complete Genome
GCF_001584185.1	na	*Collimonas pratensis*	Ter91	Complete Genome
GCF_003293745.1	na	*Herminiimonas fonticola*	S-94	Contig
GCF_004361795.1	x	*Herminiimonas fonticola*	DSM 18555	Contig
GCF_003096595.1	na	*Pusillimonas noertemannii*	DSM 10065	Scaffold
GCF_003545825.1	x	*Pusillimonas noertemannii*	DSM 10065	Contig
GCF_013416295.1	na	*Pusillimonas noertemannii*	DSM 10065	Contig
GCF_007556675.1	na	*Tepidimonas taiwanensis*	I1-1	Contig
GCF_020162115.1	x	*Tepidimonas taiwanensis*	LMG 22826	Complete Genome
GCF_002902925.1	na	*Paraburkholderia terrae*	DSM 17804	Complete Genome
GCF_902833725.1	na	*Paraburkholderia mimosarum*	LMG 23256	Scaffold
GCF_000016345.1	x	*Polynucleobacter asymbioticus*	QLW-P1DMWA-1	Complete Genome
GCF_009727155.1	x	*Massilia dura*	DSM 17513	Contig
GCF_014651395.1	na	*Massilia dura*	KCTC 12342	Scaffold
GCF_004322755.1	x	*Massilia albidiflava*	DSM 17472	Complete Genome
GCF_014651415.1	na	*Massilia albidiflava*	KCTC 12343	Scaffold
GCF_004421005.1	x	*Massilia plicata*	DSM 17505	Complete Genome
GCF_014651435.1	na	*Massilia plicata*	KCTC 12344	Scaffold
GCF_004209755.1	x	*Massilia lutea*	DSM 17473	Chromosome
GCF_003063475.1	x	*Limnohabitans curvus*	MWH-C5	Contig
GCF_001544475.1	x	*Caballeronia humi*		Contig
GCF_001544495.1	x	*Caballeronia telluris*		Contig
GCF_001544535.1	x	*Caballeronia choica*		Contig
GCF_007556705.1	x	*Tepidimonas thermarum*	AA-1	Contig
GCF_000013605.1	x	*Rhodoferax ferrireducens T118*	DSM 15236	Complete Genome
GCF_000203915.1	na	*Burkholderia ambifaria AMMD*	AMMD	Complete Genome

Assembly accession	Representative genome	Organism name	Type strain name	Assembly level
GCF_000959545.1	na	*Burkholderia ambifaria AMMD*	AMMD	Complete Genome
GCF_001418255.1	x	*Thiomonas bhubaneswarensis*	DSM 18181	Scaffold
GCF_001517285.1	na	*Thiomonas bhubaneswarensis*	DSM 18181	Scaffold
GCF_900608545.1	na	*Burkholderia oklahomensis*	LMG 23618	Contig
GCF_902829515.1	na	*Burkholderia oklahomensis*	LMG 23618	Scaffold
GCF_001298675.1	na	*Acidovorax caeni*	R-24608	Contig
GCF_900116825.1	x	*Acidovorax caeni*	R-24608	Contig
GCF_001189965.1	x	*Herbaspirillum rhizosphaerae*	UMS-37	Contig
GCF_002224225.1	x	*Polynucleobacter cosmopolitanus*	MWH-MoIso2	Contig
GCF_004363315.1	x	*Tepidicella xavieri*	DSM 19605	Scaffold
GCF_000964545.1	na	*Comamonas thiooxydans*	DSM 17888	Contig
GCF_001418295.1	na	*Comamonas thiooxydans*	DSM 17888	Scaffold
GCF_001517325.1	na	*Comamonas thiooxydans*	DSM 17888	Scaffold
GCF_900088825.1	x	*Thiomonas delicata*	DSM 16361	Scaffold
GCF_000015505.1	x	*Polaromonas naphthalenivorans CJ2*	CJ2	Complete Genome
GCF_000215705.1	x	*Ramlibacter tataouinensis TTB310*	TTB310	Complete Genome
GCF_001854325.1	x	*Cupriavidus malaysiensis*	USMAA1020	Complete Genome
GCF_002879855.1	na	*Trinickia soli*	GP25-8	Contig
GCF_902859815.1	x	*Trinickia soli*	LMG 24076	Contig
GCF_013416525.1	na	*Parapusillimonas granuli*	LMG 24012	Contig
GCF_014202705.1	x	*Parapusillimonas granuli*	DSM 18079	Scaffold
GCF_000020045.1	x	*Paraburkholderia phymatum STM815*	STM815	Complete Genome
GCF_902833665.1	na	*Paraburkholderia phymatum STM815*	LMG 21445	Scaffold
GCF_000015565.1	x	*Verminephrobacter eiseniae EF01-2*	EF01-2	Complete Genome
GCF_005952805.1	x	*Inhella inkyongensis*	IMCC1713	Complete Genome
GCF_014202795.1	na	*Inhella inkyongensis*	DSM 23958	Scaffold
GCF_000172415.1	na	*Paraburkholderia graminis C4D1M*	C4D1M	Contig

Assembly accession	Representative genome	Organism name	Type strain name	Assembly level
GCF_902859785.1	na	*Paraburkholderia graminis C4D1M*	LMG 18924	Contig
GCF_001548475.1	na	*Bordetella hinzii LMG 13501*	LMG 13501	Contig
GCF_000020125.1	x	*Paraburkholderia phytofirmans*	PsJN	Complete Genome
GCF_003201815.1	x	*Undibacterium pigrum*	DSM 19792	Scaffold
GCF_003955735.1	x	*Undibacterium parvum*	DSM 23061	Complete Genome
GCF_001017435.1	x	*Schlegelella brevitalea*	DSM 7029	Complete Genome
GCF_900104845.1	x	*Paraburkholderia caballeronis*	TNe-841	Contig
GCF_900109675.1	na	*Paraburkholderia caballeronis*	LMG 26416	Scaffold
GCF_900096975.1	x	*Paraburkholderia lycopersici*	TNe-862	Contig
GCF_002927045.1	x	*Mycetohabitans endofungorum*	HKI456	Contig
GCF_000015725.1	x	*Methylibium petroleiphilum PM1*	PM1	Complete Genome
GCF_003269035.1	x	*Paraburkholderia bryophila*	LMG 23644	Scaffold
GCF_007556815.1	na	*Paraburkholderia megapolitana*	LMG 23650	Complete Genome
GCF_900113825.1	x	*Paraburkholderia megapolitana*	LMG 23650	Contig
GCF_000253115.1	x	*Thiomonas arsenitoxydans*	3As	Chromosome
GCF_002205845.1	x	*Pelomonas puraquae*	CCUG 52769	Contig
GCF_014192155.1	x	*Roseateles terrae*	CECT 7247	Scaffold
GCF_002205645.1	x	*Roseateles aquatilis*	CCUG 48205	Contig
GCF_003350475.1	x	*Pseudacidovorax intermedius*	DSM 21352	Scaffold
GCF_000170375.1	na	*Burkholderia oklahomensis C6786*	C6786	Contig
GCF_000959365.1	x	*Burkholderia oklahomensis C6786*	C6786	Complete Genome
GCF_001522135.2	na	*Burkholderia oklahomensis C6786*	C6786	Complete Genome
GCF_001017775.3	x	*Pandoraea thiooxydans*	DSM 25325	Complete Genome
GCF_001931675.1	na	*Pandoraea thiooxydans*	ATSB16	Complete Genome
GCF_009909205.1	x	*Aquabacterium fontiphilum*	CS-6	Contig
GCF_004153455.1	x	*Pusillimonas ginsengisoli*	KCTC 22046	Contig
GCF_004217215.1	x	*Rivibacter subsaxonicus*	DSM 19570	Scaffold

Assembly accession	Representative genome	Organism name	Type strain name	Assembly level
GCF_902859805.1	x	*Paraburkholderia sediminicola*	LMG 24238	Contig
GCF_001676725.1	x	*Bordetella flabilis*	AU10664	Complete Genome
GCF_001676705.1	na	*Bordetella bronchialis*	AU3182	Complete Genome
GCF_002209535.1	na	*Achromobacter marplatensis*	B2	Contig
GCF_003315095.1	na	*Achromobacter marplatensis*	CECT 7342	Scaffold
GCF_902859635.1	x	*Achromobacter marplatensis*	LMG 26219	Contig
GCF_000012945.1	x	*Burkholderia lata*	383	Complete Genome
GCF_002879865.1	na	*Paraburkholderia rhynchosiae*	WSM 3937	Contig
GCF_902859775.1	x	*Paraburkholderia rhynchosiae*	LMG 27174	Contig
GCF_008802135.1	na	*Burkholderia latens*	CCUG 54555	Contig
GCF_902499045.1	na	*Burkholderia latens*		Contig
GCF_001758385.2	na	*Burkholderia contaminans*	LMG 23361	Contig
GCF_902499065.1	na	*Burkholderia metallica*		Contig
GCF_902832845.1	na	*Burkholderia metallica*	LMG 24068	Scaffold
GCF_902499125.1	na	*Burkholderia arboris*		Contig
GCF_902499165.1	na	*Burkholderia seminalis*		Contig
GCF_008802145.1	na	*Burkholderia diffusa*	CCUG 54558	Contig
GCF_016836765.1	na	*Burkholderia diffusa*	DSM 23434	Contig
GCF_902499115.1	na	*Burkholderia diffusa*		Contig
GCF_902833905.1	na	*Paraburkholderia tuberum STM678*	LMG 21444	Scaffold
GCF_006519715.1	na	*Ideonella azotifigens*	DSM 21438	Contig
GCF_014651835.1	x	*Pigmentiphaga litoralis*	KCTC 22165	Scaffold
GCF_013410775.1	x	*Sphaerotilus montanus*	DSM 21226	Contig
GCF_013426955.1	na	*Sphaerotilus montanus*	HS	Contig
GCF_003337555.1	x	*Pseudorhodoferax soli*	DSM 21634	Scaffold
GCF_900143065.1	x	*Duganella sacchari*	Sac-22	Scaffold
GCF_009720825.1	x	*Duganella radicis*	KCTC 22382	Contig

Assembly accession	Representative genome	Organism name	Type strain name	Assembly level
GCF_002157145.1	na	*Acidovorax carolinensis*	NA3	Complete Genome
GCF_003065385.1	x	*Polynucleobacter acidiphobus*	MWH-PoolGreenA3	Chromosome
GCF_003065365.1	x	*Polynucleobacter difficilis*	AM-8B5	Complete Genome
GCF_003072505.1	x	*Polynucleobacter rarus*	MT-CBb6A5	Scaffold
GCF_900107225.1	na	*Delftia lacustris*	LMG 24775	Contig
GCF_002879885.1	x	*Trinickia dabaoshanensis*	GIMN1.004	Contig
GCF_000193225.1	x	*Verminephrobacter aporrectodeae subsp. tuberculatae*	At4	Contig
GCF_000306945.1	na	*Bordetella pertussis 18323*	18323	Complete Genome
GCF_002213425.1	x	*Herbaspirillum aquaticum*	IEH 4430	Contig
GCF_000972785.3	x	*Pandoraea oxalativorans*	DSM 23570	Complete Genome
GCF_014635705.1	x	*Oxalicibacterium faecigallinarum*	CCM 2767	Scaffold
GCF_904848625.1	x	*Paraburkholderia metrosideri*	LMG 28140	Contig
GCF_004362405.1	x	*Kinneretia asaccharophila*	DSM 25082	Scaffold
GCF_900107605.1	x	*Acidovorax soli*	DSM 25157	Contig
GCF_000204645.1	na	*Alicycliphilus denitrificans K601*	K601	Complete Genome
GCF_000498435.1	na	*Rhodoferax saidenbachensis ED16*	ED16	Contig
GCF_902859895.1	x	*Pararobbsia alpina*	LMG 28138	Contig
GCF_004346225.1	x	*Paralcaligenes ureilyticus*	DSM 24591	Contig
GCF_013426975.1	na	*Sphaerotilus sulfidivorans*	D-501	Contig
GCF_000176855.2	x	*Acidovorax avenae subsp. avenae*	ATCC 19860	Complete Genome
GCF_011927625.1	x	*Paenalcaligenes hominis*	DSM 26613	Scaffold
GCF_014635265.1	na	*Paenalcaligenes hominis*	CCM 7698	Contig
GCF_003315175.1	na	*Eoetvoesia caeni*	DSM 25520	Scaffold
GCF_013416545.1	x	*Eoetvoesia caeni*	PB3-7B	Contig
GCF_022688825.1	na	*Eoetvoesia caeni*	PB3-7B	Contig
GCF_000934605.2	x	*Pandoraea vervacti*	NS15	Complete Genome
GCF_001029105.3	x	*Pandoraea faecigallinarum*	DSM 23572	Complete Genome

Assembly accession	Representative genome	Organism name	Type strain name	Assembly level
GCF_004153685.1	x	*Pusillimonas harenae*	JCM 16917	Contig
GCF_013416515.1	na	*Pusillimonas harenae*	DSM 25667	Contig
GCF_900129885.1	x	*Candidimonas bauzanensis*	CGMCC 1.10190	Scaffold
GCF_004153445.1	x	*Pusillimonas soli*	KCTC 22455	Scaffold
GCF_013416335.1	na	*Pusillimonas soli*	DSM 25264	Contig
GCF_013377855.1	x	*Undibacterium oligocarboniphilum*	EM 1	Scaffold
GCF_900108945.1	x	*Paraburkholderia diazotrophica*	LMG 26031	Scaffold
GCF_001544075.1	x	*Comamonas terrae*	NBRC 106524	Contig
GCF_002209565.1	x	*Candidimonas nitroreducens*	SC-089	Contig
GCF_019166065.1	x	*Candidimonas humi*	DSM 25336	Contig
GCF_000818395.1	x	*Noviherbaspirillum autotrophicum*	TSA66	Scaffold
GCF_011947285.1	x	*Glaciimonas immobilis*	DSM 23240	Scaffold
GCF_014202815.1	na	*Glaciimonas immobilis*	DSM 23240	Scaffold
GCF_000262525.1	x	*Leptothrix ochracea L12*	L12	Scaffold
GCF_000276685.1	na	*Taylorella equigenitalis ATCC 35865*	ATCC 35865	Complete Genome
GCF_900637125.1	na	*Taylorella equigenitalis ATCC 35865*	NCTC 11184	Complete Genome
GCF_003201595.1	x	*Sphaerotilus hippei*	DSM 566	Scaffold
GCF_014652235.1	x	*Pseudorhodoferax aquiterrae*	KCTC 23314	Scaffold
GCF_000473465.1	na	*Paraburkholderia sprentiae*	WSM5005	Scaffold
GCF_001865575.2	x	*Paraburkholderia sprentiae*	WSM5005	Complete Genome
GCF_000402035.1	x	*Caballeronia insecticola*	RPE64	Complete Genome
GCF_900114705.1	x	*Rugamonas rubra*	ATCC 43154	Scaffold
GCF_001758785.1	x	*Duganella phyllosphaerae*	T54	Contig
GCF_000205025.1	x	*Parasutterella excrementihominis*	YIT 11859	Scaffold
GCF_000250875.1	x	*Sutterella parvirubra YIT 11816*	YIT 11816	Scaffold
GCF_002105195.1	x	*Derxia lacustris*	HL-12	Contig
GCF_014652395.1	x	*Advenella faeciporci*	KCTC 23732	Contig

Assembly accession	Representative genome	Organism name	Type strain name	Assembly level
GCF_002934455.1	na	*Trinickia symbiotica*	JPY-345	Scaffold
GCF_000300975.2	x	*Herbaspirillum frisingense GSF30*	GSF30	Contig
GCF_014192225.1	na	*Massilia umbonata*	CECT 7753	Scaffold
GCF_000698575.1	na	*Caballeronia zhejiangensis*	OP-1	Contig
GCF_007830495.1	na	*Massilia flava*	CGMCC 1.10685	Scaffold
GCF_000198775.1	x	*Mycetohabitans rhizoxinica HKI 454*	HKI 454	Complete Genome
GCF_000315425.1	na	*Massilia timonae CCUG 45783*	CCUG 45783	Scaffold
GCF_000211835.1	x	*Hylemonella gracilis ATCC 19624*	ATCC 19624	Contig
GCF_000186425.1	na	*Lautropia mirabilis ATCC 51599*	ATCC 51599	Scaffold
GCF_001028175.1	x	*Caballeronia mineralivorans*	PML1(12)	Contig
GCF_905397285.1	na	*Cupriavidus plantarum*	LMG 26296	Contig
GCF_900094595.1	na	*Cupriavidus alkaliphilus*	ASC-732	Scaffold
GCF_003143515.1	x	*Massilia oculi*	CCUG 43427	Complete Genome
GCF_002116905.1	na	*Rhizobacter gummiphilus*	NS21	Complete Genome
GCF_002762215.1	x	*Rhizobacter gummiphilus*	NBRC 109400	Complete Genome
GCF_000472525.1	x	*Paraburkholderia dilworthii*	WSM3556	Scaffold
GCF_000069785.1	x	*Cupriavidus taiwanensis LMG 19424*	LMG 19424	Chromosome
GCF_000473485.1	na	*Burkholderia cepacia ATCC 25416*	ATCC 25416	Scaffold
GCF_001411495.1	na	*Burkholderia cepacia ATCC 25416*	UCB 717	Complete Genome
GCF_003546465.1	na	*Burkholderia cepacia ATCC 25416*	25416	Complete Genome
GCF_006094315.1	na	*Burkholderia cepacia ATCC 25416*	ATCC 25416	Complete Genome
GCF_000286555.1	na	*Burkholderia multivorans*	ATCC BAA-247	Contig
GCF_000959525.1	na	*Burkholderia multivorans*	BAA-247	Complete Genome
GCF_000190375.1	na	*Rubrivivax benzoatilyticus*	JA2	Contig
GCF_000420125.1	na	*Rubrivivax benzoatilyticus*	JA2	Contig
GCF_000204195.1	x	*Acidovorax radicis N35*	N35	Contig
GCF_004217045.1	x	*Cupriavidus agavae*	ASC-9842	Contig

Assembly accession	Representative genome	Organism name	Type strain name	Assembly level
GCF_000241525.1	na	*Comamonas testosteroni*	ATCC 11996	Contig
GCF_003337425.1	x	*Extensimonas vulgaris*	DSM 100911	Scaffold
GCF_007830535.1	na	*Extensimonas vulgaris*	CGMCC 1.10977	Scaffold
GCF_007996985.1	na	*Extensimonas vulgaris*	S4	Scaffold
GCF_900116645.1	x	*Massilia namucuonensis*	CGMCC 1.11014	Scaffold
GCF_007830455.1	x	*Massilia lurida*	CGMCC 1.10822	Contig
GCF_000219915.2	x	*Advenella kashmirensis WT001*	WT001	Complete Genome
GCF_000472825.1	x	*Paraburkholderia mimosarum*	LMG 23256	Scaffold
GCF_000739815.1	na	*Paraburkholderia mimosarum*	NBRC 106338	Contig
GCF_000372525.1	na	*Cupriavidus neocaledonicus*	STM6070	Contig
GCF_000219215.1	na	*Cupriavidus necator N-1*	N-1	Complete Genome
GCF_015352955.1	x	*Noviherbaspirillum soli*	SUEMI10	Contig
GCF_000698555.1	na	*Caballeronia grimmiae*	R27	Contig
GCF_014636375.1	na	*Caballeronia grimmiae*	CGMCC 1.11013	Contig
GCF_000743945.1	x	*Basilea psittacipulmonis DSM 24701*	DSM 24701	Complete Genome
GCF_002003525.1	na	*Basilea psittacipulmonis DSM 24701*	DSM 24701	Contig
GCF_003703475.1	x	*Franklinella schreckenbergeri*	NML 97-0147	Contig
GCF_904848665.1	x	*Paraburkholderia hiiakae*	LMG 27952	Contig
GCF_002158865.1	x	*Comamonas serinivorans*	DSM 26136	Complete Genome
GCF_002251695.1	na	*Ralstonia solanacearum K60*	K60	Contig
GCF_000312045.1	x	*Noviherbaspirillum massiliense*	JC206	Scaffold
GCF_007556755.1	na	*Tepidimonas fonticaldi*	AT-A2	Contig
GCF_001883705.2	x	*Burkholderia catarinensis*	89	Contig
GCF_902832885.1	na	*Burkholderia seminalis DSM 23518*	LMG 24067	Scaffold
GCF_001938565.1	na	*Rhodoferax antarcticus ANT.BR*	ANT.BR	Contig
GCF_000427785.1	x	*Massilia alkalitolerans DSM 17462*	DSM 17462	Scaffold
GCF_014652415.1	x	*Undibacterium macrobrachii*	KCTC 23916	Contig

Assembly accession	Representative genome	Organism name	Type strain name	Assembly level
GCF_000430725.1	x	*Azohydromonas australica*	DSM 1124	Scaffold
GCF_000374625.1	x	*Brachymonas chironomi DSM 19884*	DSM 19884	Scaffold
GCF_900110225.1	x	*Brachymonas denitrificans*	DSM 15123	Scaffold
GCF_000621025.1	x	*Brackiella oedipodis DSM 13743*	DSM 13743	Scaffold
GCF_000519185.1	x	*Paraburkholderia nodosa*	DSM 21604	Scaffold
GCF_009646335.1	x	*Caenimonas koreensis DSM 17982*	EMB320	Contig
GCF_000428465.1	x	*Chitinimonas koreensis DSM 17726*	DSM 17726	Scaffold
GCF_900119825.1	x	*Chitinimonas taiwanensis*	DSM 18899	Scaffold
GCF_000484635.1	x	*Comamonas badia DSM 17552*	DSM 17552	Contig
GCF_000429845.1	x	*Comamonas composti DSM 21721*	DSM 21721	Scaffold
GCF_000482785.1	x	*Derxia gummosa DSM 723*	DSM 723	Scaffold
GCF_000425385.1	x	*Pseudoduganella violaceinigra*	DSM 15887	Scaffold
GCF_900129055.1	x	*Lampropedia hyalina DSM 16112*	DSM 16112	Scaffold
GCF_000382345.1	x	*Massilia niastensis DSM 21313*	DSM 21313	Scaffold
GCF_000373745.1	x	*Oligella ureolytica DSM 18253*	DSM 18253	Scaffold
GCF_000372065.1	na	*Oligella urethralis DSM 7531*	DSM 7531	Contig
GCF_000422885.1	x	*Ottowia thiooxydans DSM 14619*	DSM 14619	Scaffold
GCF_000688255.1	x	*Simplicispira psychrophila*	DSM 11588	Contig
GCF_000745855.1	x	*Xenophilus azovorans DSM 13620*	DSM 13620	Scaffold
GCF_014652435.1	x	*Undibacterium squillarum*	KCTC 23917	Contig
GCF_001941825.1	x	*Massilia putida*	6NM-7	Complete Genome
GCF_000256565.1	x	*Herbaspirillum lusitanum P6-12*	P6-12	Contig
GCF_900112225.1	x	*Massilia yuzhufengensis*	CGMCC 1.12041	Scaffold
GCF_000282995.1	na	*Melaminivora alkalimesophila*	CY1	Contig
GCF_003182375.1	x	*Melaminivora alkalimesophila*	DSM 26006	Scaffold
GCF_000960995.1	na	*Burkholderia glumae*	ATCC 33617	Complete Genome
GCF_902832765.1	na	*Burkholderia glumae*	LMG 2196	Scaffold

Assembly accession	Representative genome	Organism name	Type strain name	Assembly level
GCF_900113035.1	x	*Acidovorax wautersii*	DSM 27981	Scaffold
GCF_002917095.1	na	*Paraburkholderia eburnea*	JCM 18070	Scaffold
GCF_902859935.1	x	*Paraburkholderia fynbosensis*	LMG 27177	Contig
GCF_902499075.1	na	*Burkholderia pseudomultivorans*		Contig
GCF_004402975.1	x	*Paraburkholderia dipogonis*	ICMP 19430	Contig
GCF_014637085.1	x	*Comamonas phosphati*	CGMCC 1.12294	Scaffold
GCF_001598595.1	na	*Achromobacter xylosoxidans*	NBRC 15126	Contig
GCF_001571245.1	na	*Achromobacter piechaudii*	NBRC 102461	Contig
GCF_001598655.1	na	*Bordetella bronchiseptica*	NBRC 13691	Contig
GCF_000684975.1	x	*Paraburkholderia acidipaludis*	NBRC 101816	Contig
GCF_000685015.1	x	*Paraburkholderia bannensis*	NBRC 103871	Contig
GCF_000685035.1	x	*Paraburkholderia ferrariae*	NBRC 106233	Contig
GCF_000685055.1	na	*Paraburkholderia fungorum*	NBRC 102489	Contig
GCF_000739735.1	na	*Paraburkholderia ginsengisoli*	NBRC 100965	Contig
GCF_000739755.1	na	*Burkholderia gladioli*	NBRC 13700	Contig
GCF_000739775.1	x	*Paraburkholderia heleia*	NBRC 101817	Contig
GCF_000739795.1	na	*Paraburkholderia kururiensis subsp. thiooxydans*	NBRC 107107	Contig
GCF_000685075.1	x	*Paraburkholderia oxyphila*	NBRC 105797	Contig
GCF_000739835.1	na	*Paraburkholderia terrae*	NBRC 100964	Contig
GCF_000739855.1	na	*Alcaligenes faecalis subsp. faecalis*	NBRC 13111	Contig
GCF_001748345.1	na	*Alcaligenes faecalis subsp. faecalis*	NBRC 13111	Contig
GCF_001598755.1	na	*Cupriavidus necator NBRC 102504*	NBRC 102504	Contig
GCF_001598795.1	na	*Delftia acidovorans NBRC 14950*	NBRC 14950	Contig
GCF_001544155.1	na	*Ralstonia pickettii NBRC 102503*	NBRC 102503	Contig
GCF_000685095.1	na	*Paraburkholderia caledonica*	NBRC 102488	Contig
GCF_000739875.1	na	*Comamonas aquatica NBRC 14918*	NBRC 14918	Contig
GCF_001571325.1	na	*Delftia tsuruhatensis NBRC 16741*	NBRC 16741	Contig

Assembly accession	Representative genome	Organism name	Type strain name	Assembly level
GCF_001591225.1	na	*Herbaspirillum rubrisubalbicans*	NBRC 102523	Contig
GCF_000739995.1	x	*Comamonas granuli NBRC 101663*	NBRC 101663	Contig
GCF_014636315.1	x	*Undibacterium terreum*	CGMCC 1.10998	Contig
GCF_001544515.1	x	*Caballeronia terrestris*		Contig
GCF_001544555.2	x	*Caballeronia udeis*		Contig
GCF_018069865.1	x	*Ideonella paludis*	KCTC 32238	Contig
GCF_000478365.1	na	*Herbaspirillum huttiense subsp. putei*	IAM 15032	Contig
GCF_003986935.1	na	*Paraburkholderia kururiensis*	KP23	Contig
GCF_902833705.1	na	*Paraburkholderia kururiensis*	LMG 19447	Contig
GCF_001636975.1	na	*Paraburkholderia kirstenboschensis*	KB15	Contig
GCF_904848585.1	x	*Paraburkholderia kirstenboschensis*	LMG 28727	Contig
GCF_007558815.1	x	*Verticiella sediminum*	DSM 27279	Contig
GCF_000521505.1	x	*Advenella mimigardefordensis DPN7*	DPN7	Complete Genome
GCF_900019265.1	x	*Paraburkholderia ribeironis*	STM 7296	Scaffold
GCF_001571365.1	na	*Achromobacter denitrificans*	NBRC 15125	Contig
GCF_015352985.1	x	*Noviherbaspirillum malthae*	CC-AFH3	Contig
GCF_001267925.1	x	*Herbaspirillum hiltneri N3*	N3	Complete Genome
GCF_000381125.1	x	*Caldimonas manganoxidans*	ATCC BAA-369	Scaffold
GCF_000381265.1	x	*Curvibacter lanceolatus*	ATCC 14669	Scaffold
GCF_017811175.1	na	*Melaminivora jejuensis*	KCTC 32230	Contig
GCF_001592305.1	x	*Hydrogenophaga taeniospiralis*	NBRC 102512	Contig
GCF_000689195.1	na	*Sphaerotilus natans subsp. natans*	DSM 6575	Contig
GCF_902859645.1	na	*Achromobacter insuavis*	LMG 26845	Contig
GCF_902859735.1	na	*Achromobacter aegrifaciens*	LMG 26852	Contig
GCF_903652925.1	x	*Achromobacter anxifer*	LMG 26857	Contig
GCF_902859745.1	na	*Achromobacter dolens*	LMG 26840	Contig
GCF_014489535.1	x	*Diaphorobacter aerolatus*	KACC 16536	Complete Genome

Assembly accession	Representative genome	Organism name	Type strain name	Assembly level
GCF_001270065.2	x	*Limnohabitans planktonicus II-D5*	II-D5	Contig
GCF_003063455.1	x	*Limnohabitans parvus II-B4*	II-B4	Contig
GCF_003100395.1	x	*Aquabacterium olei*	NBRC 110486	Complete Genome
GCF_919586305.1	na	*Ralstonia pseudosolanacearum*	LMG 9673	Contig
GCF_001571085.1	x	*Azohydromonas lata NBRC 102462*	NBRC 102462	Contig
GCF_001598235.1	x	*Polaromonas jejuensis*	NBRC 106434	Contig
GCF_001591345.1	na	*Variovorax boronicumulans*	NBRC 103145	Contig
GCF_001591365.1	x	*Variovorax paradoxus NBRC 15149*	NBRC 15149	Contig
GCF_001591385.1	x	*Variovorax soli NBRC 106424*	NBRC 106424	Contig
GCF_900116445.1	na	*Paraburkholderia aspalathi*	LMG 27731	Scaffold
GCF_004833285.1	x	*Lampropedia puyangensis*	2-bin	Scaffold
GCF_000657795.2	na	*Bordetella pseudohinzii*	8-296-03	Contig
GCF_000987075.1	na	*Burkholderia contaminans*	LMG 23361	Contig
GCF_000518645.1	x	*Curvibacter gracilis ATCC BAA-807*	ATCC BAA-807	Scaffold
GCF_001598255.1	x	*Mitsuaria chitosanitabida*	NBRC 102408	Contig
GCF_014284255.1	x	*Undibacterium jejuense*	KACC 12607	Scaffold
GCF_014284305.1	x	*Undibacterium seohonense*	KACC 16656	Scaffold
GCF_003545815.1	x	*Pusillimonas caeni*	KCTC 42353	Contig
GCF_001571145.1	x	*Hydrogenophaga flava NBRC 102514*	NBRC 102514	Contig
GCF_001592245.1	na	*Cupriavidus oxalaticus NBRC 13593*	NBRC 13593	Contig
GCF_001592265.1	x	*Curvibacter delicatus NBRC 14919*	NBRC 14919	Contig
GCF_001571165.1	x	*Herbaspirillum chlorophenolicum*	NBRC 102525	Contig
GCF_000723165.1	x	*Janthinobacterium agaricidamnosum*	DSM 9628	Complete Genome
GCF_001571185.1	na	*Janthinobacterium agaricidamnosum*	NBRC 102515	Contig
GCF_001571205.1	x	*Hydrogenophaga intermedia*	NBRC 102510	Contig
GCF_001571225.1	x	*Hydrogenophaga palleronii*	NBRC 102513	Contig
GCF_001592285.1	na	*Hydrogenophaga pseudoflava*	NBRC 102511	Contig

Assembly accession	Representative genome	Organism name	Type strain name	Assembly level
GCF_001278535.1	na	*Caballeronia cordobensis*	LMG 27620	Scaffold
GCF_001544575.2	x	*Caballeronia cordobensis*		Contig
GCF_900496965.1	x	*Achromobacter agilis*	LMG 3411	Contig
GCF_902859625.1	x	*Achromobacter pestifer*	LMG 3431	Contig
GCF_902859595.1	na	*Achromobacter kerstersii*	LMG 3441	Contig
GCF_902859705.1	x	*Achromobacter deleyi*	LMG 3458	Contig
GCF_014652675.1	x	*Formosimonas limnophila*	KCTC 32501	Contig
GCF_001523745.2	na	*Burkholderia mayonis*	BDU6	Complete Genome
GCF_902859725.1	na	*Achromobacter mucicolens*	LMG 26685	Contig
GCF_902859765.1	na	*Achromobacter pulmonis*	LMG 26696	Contig
GCF_902859585.1	na	*Achromobacter animicus*	LMG 26690	Contig
GCF_001580545.1	x	*Paraburkholderia monticola*	JC2948	Contig
GCF_000687165.1	x	*Acidovorax oryzae ATCC 19882*	ATCC 19882	Scaffold
GCF_000687755.1	na	*Taylorella asinigenitalis*	ATCC 700933	Contig
GCF_000506865.1	x	*Pelistega indica*	HM-7	Contig
GCF_003002115.1	x	*Paraburkholderia insulsa*	LMG 28183	Contig
GCF_000959445.1	na	*Burkholderia vietnamiensis*	LMG 10929	Complete Genome
GCF_002930615.1	x	*Zhizhongheella caldifontis*	BCRC 80649	Contig
GCF_000828895.1	x	*Serpentinimonas raichei*	A1	Complete Genome
GCF_000828915.1	x	*Serpentinimonas maccroryi*	B1	Complete Genome
GCF_000696225.1	x	*Serpentinimonas barnesii*	H1	Scaffold
GCF_009720865.1	x	*Massilia ginsengisoli*	KCTC 42409	Contig
GCF_001645125.1	na	*Paraburkholderia ginsengiterrae*	DCY85	Contig
GCF_001645135.1	x	*Paraburkholderia ginsengiterrae*	DCY85-1	Contig
GCF_019132845.1	x	*Advenella alkanexedens*	LAM0050	Contig
GCF_014652815.1	x	*Alcaligenes pakistanensis*	KCTC 42083	Scaffold
GCF_001955715.1	x	*Rhodoferax saidenbachensis*	DSM 22694	Complete Genome

Assembly accession	Representative genome	Organism name	Type strain name	Assembly level
GCF_002760655.1	x	*Massilia eurypsychrophila*	JCM 30074	Contig
GCF_900176645.1	na	*Burkholderia singularis*	LMG 28154	Contig
GCF_008802125.1	na	*Burkholderia stagnalis*	CCUG 65686	Contig
GCF_902499155.1	na	*Burkholderia stagnalis*		Contig
GCF_902830275.1	na	*Burkholderia stagnalis*	LMG 28156	Contig
GCF_008802115.1	na	*Burkholderia territorii*	CCUG 65687	Contig
GCF_902499035.1	na	*Burkholderia territorii*		Contig
GCF_902833055.1	na	*Burkholderia territorii*	LMG 28158	Contig
GCF_001182045.1	x	*Dakarella massiliensis*	ND3	Contig
GCF_000732615.1	na	*Burkholderia paludis*	MSh1	Contig
GCF_003011895.2	x	*Massilia glaciei*	B448-2	Contig
GCF_900103645.1	x	*Oryzisolibacter propanilivorax*	EPL6	Contig
GCF_004363775.1	x	*Hydromonas duriensis*	DSM 102852	Scaffold
GCF_014699135.1	x	*Undibacterium aquatile*	CCTCC AB 2015119	Scaffold
GCF_900258035.1	x	*Caballeronia novacaledonica*	LMG 28615	Contig
GCF_902859915.1	x	*Paraburkholderia ultramafica*	LMG 28614	Contig
GCF_001293525.1	x	*Ideonella sakaiensis*	201-F6	Contig
GCF_014202215.1	x	*Quisquiliibacterium transsilvanicum*	DSM 29781	Scaffold
GCF_004358105.1	x	*Sapientia aquatica*	SA-152	Scaffold
GCF_003703815.1	x	*Corticibacter populi*	DSM 105136	Contig
GCF_004217315.1	na	*Corticibacter populi*	DSM 105136	Scaffold
GCF_003966915.1	x	*Mycoavidus cysteinexigens*	B1EB	Complete Genome
GCF_002760665.1	x	*Massilia psychrophila*	JCM 30813	Contig
GCF_014641415.1	na	*Massilia psychrophila*	CGMCC 1.15196	Contig
GCF_001005215.1	x	*Lampropedia cohaerens*	CT6	Contig
GCF_014641715.1	x	*Polaromonas eurypsychrophila*	CGMCC 1.15322	Scaffold
GCF_013133795.1	x	*Pelistega suis*	3340-03	Contig

Assembly accession	Representative genome	Organism name	Type strain name	Assembly level
GCF_003965815.1	x	*Variovorax gossypii*	DSM 100435	Contig
GCF_900188095.1	x	*Noviherbaspirillum humi*	U15	Scaffold
GCF_900007165.1	x	*Paraburkholderia piptadeniae*	STM 7183	Scaffold
GCF_014191875.1	x	*Massilia violacea*	CECT 8897	Contig
GCF_014395975.1	x	*Diaphorobacter ruginosibacter*	DSM 27467	Complete Genome
GCF_902859945.1	x	*Paraburkholderia caffeinitolerans*	LMG 28688	Contig
GCF_001595985.1	x	*Polynucleobacter sinensis*	MWH-HuW1	Scaffold
GCF_001595965.1	x	*Polynucleobacter yangtzensis*	MWH-JaK3	Scaffold
GCF_013307245.1	na	*Polynucleobacter antarcticus*	LimPoW16	Complete Genome
GCF_002206625.1	x	*Polynucleobacter campilacus*	MWH-Feld-100	Contig
GCF_002206635.1	x	*Polynucleobacter aenigmaticus*	MWH-K35W1	Contig
GCF_002192535.1	x	*Polynucleobacter hirudinilacicola*	MWH-EgelM1-30-B4	Contig
GCF_001659725.1	x	*Polynucleobacter wuianus*	QLW-P1FAT50C-4	Chromosome
GCF_001953355.1	x	*Polynucleobacter sphagniphilus*	MWH-Weng1-1	Contig
GCF_013307225.1	na	*Polynucleobacter tropicus*	MWH-UH21B	Complete Genome
GCF_001677885.1	x	*Janthinobacterium psychrotolerans*	S3-2	Contig
GCF_004121055.1	x	*Achromobacter aloeverae*	AVA-1	Contig
GCF_003368325.1	x	*Paraburkholderia caffeinilytica*	CF1	Complete Genome
GCF_014640695.1	na	*Paraburkholderia caffeinilytica*	CGMCC 1.15103	Scaffold
GCF_902859905.1	na	*Paraburkholderia caffeinilytica*	LMG 28690	Contig
GCF_001640105.1	na	*Hydrogenophaga crassostreae*	LPB0072	Contig
GCF_001761385.1	x	*Hydrogenophaga crassostreae*	LPB0072	Complete Genome
GCF_900095735.1	x	*Chitinasiproducens palmae*	JS23	Scaffold
GCF_003952165.1	x	*Variovorax guangxiensis*	DSM 27352	Contig
GCF_009720745.1	x	*Massilia eburnea*	JCM 31587	Contig
GCF_001544615.1	x	*Caballeronia concitans*		Contig
GCF_001544695.2	x	*Caballeronia arvi*		Contig

Assembly accession	Representative genome	Organism name	Type strain name	Assembly level
GCF_001544755.2	x	*Caballeronia catudaia*		Contig
GCF_001544795.2	x	*Caballeronia temeraria*		Contig
GCF_001544835.2	x	*Caballeronia fortuita*		Contig
GCF_900044055.2	x	*Caballeronia calidae*		Contig
GCF_001544875.2	x	*Caballeronia hypogeia*		Contig
GCF_001544915.2	x	*Caballeronia pedi*		Contig
GCF_001544975.2	na	*Caballeronia arationis*		Contig
GCF_001545035.1	x	*Caballeronia glebae*		Contig
GCF_001545075.1	x	*Caballeronia ptereochthonis*		Contig
GCF_004681965.1	x	*Ramlibacter rhizophilus*	CCTCC AB2015357	Contig
GCF_003628125.1	x	*Pararobbsia silviterrae*	DHC34	Scaffold
GCF_013403875.1	x	*Burkholderia guangdongensis*	DHOM02	Contig
GCF_001598055.1	x	*Cupriavidus nantongensis*	X1	Chromosome
GCF_002221595.1	na	*Turicimonas muris*	YL45	Contig
GCF_016696765.1	x	*Turicimonas muris*	YL45	Chromosome
GCF_004011805.1	x	*Piscinibacter defluvii*	SH-1	Scaffold
GCF_900130075.1	x	*Herminiimonas arsenitoxidans*	AS8	Complete Genome
GCF_018687955.1	na	*Polynucleobacter ibericus*	es-MAR-2	Chromosome
GCF_018881545.1	na	*Polynucleobacter alcilacus*	UK-Pondora-W15	Scaffold
GCF_014284275.1	x	*Undibacterium amnicola*	KCTC 52442	Scaffold
GCF_000973625.1	x	*Polynucleobacter duraquae*	MWH-MoK4	Complete Genome
GCF_001955695.1	x	*Rhodoferax koreense*	DCY-110	Complete Genome
GCF_900128485.1	x	*Duodenibacillus massiliensis*	Marseille-P2968	Scaffold
GCF_018881835.1	na	*Polynucleobacter hallstattensis*	MWH-Hall10	Contig
GCF_018881715.1	na	*Polynucleobacter parvulilacunae*	Ross1-W9	Contig
GCF_018881755.1	na	*Polynucleobacter finlandensis*	MWH-Mekk-B1	Scaffold
GCF_004016505.1	x	*Aquincola rivuli*	KYPY4	Contig

Assembly accession	Representative genome	Organism name	Type strain name	Assembly level
GCF_003335215.1	x	*Parvibium lacunae*	KMB9	Contig
GCF_009720775.1	x	*Pseudoduganella danionis*	DSM 103461	Scaffold
GCF_000807775.2	x	*Pandoraea fibrosis*	6399	Complete Genome
GCF_001929405.1	x	*Herbaspirillum camelliae*	WT00C	Scaffold
GCF_002099195.1	na	*Burkholderia puraquae*	CAMPA 1040	Contig
GCF_902859845.1	x	*Burkholderia puraquae*	LMG 29660	Contig
GCF_002362315.1	x	*Paraburkholderia acidicola*	ATCC 31363	Contig
GCF_004342005.1	x	*Paracandidimonas soli*	DSM 100048	Scaffold
GCF_003843835.1	x	*Tibeticola sediminis*	DSM 101684	Scaffold
GCF_014489595.1	x	*Acidovorax monticola*	KACC 19171	Complete Genome
GCF_016722785.1	x	*Ramlibacter monticola*	KACC 19175	Scaffold
GCF_014397785.1	x	*Paraburkholderia podalyriae*	WC7.3b	Contig
GCF_900205755.1	x	*Polynucleobacter meluiroseus*	AP-Melu-1000-B4	Contig
GCF_002797575.1	na	*Polynucleobacter brandtiae*	UB-Domo-W1	Contig
GCF_900176405.1	na	*Polynucleobacter kasalickyi*	VK13	Contig
GCF_018304965.1	x	*Collimonas antrihumi*	DSM 104040	Contig
GCF_009720835.1	na	*Massilia buxea*	KCTC 52429	Scaffold
GCF_014644155.1	x	*Massilia buxea*	CGMCC 1.15931	Scaffold
GCF_003991585.1	x	*Saezia sanguinis*	CNM695-12	Contig
GCF_002211445.1	x	*Noviherbaspirillum denitrificans*	TSA40	Scaffold
GCF_002245625.1	x	*Acidovorax kalamii*	KNDSW-TSA6	Scaffold
GCF_002213415.1	na	*Herbaspirillum robiniae*	HZ10	Contig
GCF_902499175.1	na	*Burkholderia aenigmatica*	LMG 13014	Contig
GCF_002968015.1	x	*Massilia phosphatilytica*	12-OD1	Contig
GCF_002278075.1	x	*Paraburkholderia aromaticivorans*	BN5	Complete Genome
GCF_003576595.1	x	*Pusillimonas maritima*	17-4A	Scaffold
GCF_004022565.1	x	*Pusillimonas thiosulfatoxidans*	YE3	Complete Genome

Assembly accession	Representative genome	Organism name	Type strain name	Assembly level
GCF_002285285.1	x	*Vandammella animalimorsus*	NML 03-0146	Contig
GCF_016722765.1	x	*Ramlibacter alkalitolerans*	KACC 19305	Contig
GCF_002752675.1	x	*Massilia violaceinigra*	B2	Complete Genome
GCF_900187875.1	x	*Polynucleobacter victoriensis*	MWH-VicM1	Contig
GCF_007995085.1	x	*Paraburkholderia azotifigens*	NF 2-5-3	Contig
GCF_900496975.1	x	*Achromobacter veterisilvae*	LMG 30378	Contig
GCF_002885975.1	x	*Paucibacter aquatile*	CR182	Contig
GCF_003028855.1	x	*Massilia armeniaca*	ZMN-3	Complete Genome
GCF_018882205.1	na	*Polynucleobacter bastaniensis*	AP-Basta-1000A-D1	Contig
GCF_018688255.1	na	*Polynucleobacter corsicus*	AP-Melu-1000-A1	Complete Genome
GCF_018882155.1	na	*Polynucleobacter nymphae*	AP-Mumm-500A-B3	Contig
GCF_002976435.1	x	*Solimicrobium silvestre*	S20-91	Contig
GCF_003008535.1	x	*Ottowia oryzae*	KADR8-3	Complete Genome
GCF_003008595.1	x	*Simplicispira suum*	SC1-8	Complete Genome
GCF_003013695.1	x	*Pulveribacter suum*	SC2-7	Complete Genome
GCF_009455635.1	x	*Paraburkholderia agricolaris*	BaQS159	Contig
GCF_009455685.1	x	*Paraburkholderia hayleyella*	BhQS11	Contig
GCF_009455625.1	x	*Paraburkholderia bonniea*	BbQS859	Contig
GCF_003058465.1	x	*Orrella marina*	HZ20	Complete Genome
GCF_003123725.1	x	*Corticimicrobacter populi*	3d-2-2	Contig
GCF_003415675.1	x	*Rhodoferax lacus*	IMCC26218	Contig
GCF_003353175.1	x	*Paraburkholderia lacunae*	S27	Contig
GCF_003286395.1	x	*Paraburkholderia dokdonella*	DCT13	Complete Genome
GCF_003604195.1	x	*Simplicispira lacusdiani*	CPCC 100842	Scaffold
GCF_003294055.1	x	*Burkholderia reimsis*	BE51	Contig
GCF_003402575.1	x	*Mesosutterella multiformis*	4NBBH2	Contig
GCF_003574675.1	x	*Calidifontimicrobium sediminis*	SYSU G00088	Contig

Assembly accession	Representative genome	Organism name	Type strain name	Assembly level
GCF_007556685.1	x	*Tepidimonas charontis*	SPSP-6	Contig
GCF_003339525.1	x	*Ephemeroptericola cinctiostellae*	F02	Complete Genome
GCF_003443895.1	x	*Paraburkholderia phosphatilytica*	7QSK02	Scaffold
GCF_003367175.1	x	*Trinickia dinghuensis*	DHOM06	Scaffold
GCF_003390925.1	x	*Trinickia diaoshuihuensis*	NEAU-SY24	Contig
GCF_003417535.1	x	*Hydrogenophaga borbori*	LA-38	Contig
GCF_003837865.1	x	*Paraburkholderia dinghuensis*	DHOA04	Scaffold
GCF_005403045.1	x	*Aquabacterium pictum*	W35	Contig
GCF_003570885.1	x	*Simplicispira hankyongi*	NY-02	Contig
GCF_003569765.1	x	*Aquabacterium tepidiphilum*	YIM 730274	Scaffold
GCF_003590875.1	x	*Noviherbaspirillum cavernae*	K2R10-39	Contig
GCF_003591035.1	x	*Noviherbaspirillum saxi*	K1R23-30	Contig
GCF_003590855.1	x	*Massilia cavernae*	K1S02-61	Contig
GCF_003590835.1	x	*Noviherbaspirillum sedimenti*	K1S02-23	Contig
GCF_003628145.1	x	*Trinickia fusca*	7MK8-2	Scaffold
GCF_003892345.1	x	*Lautropia dentalis*	KCOM 2505	Scaffold
GCF_003933735.1	x	*Aquabacterium soli*	SJQ9	Contig
GCF_003609995.1	x	*Sutterella megalosphaeroides*	6FBBBH3	Complete Genome
GCF_003953935.1	x	*Massilia atriviolacea*	SOD	Contig
GCF_003951285.1	na	*Variovorax beijingensis*	502	Contig
GCF_003852895.1	x	*Albitalea terrae*	S-16	Contig
GCF_008039575.1	x	*Zeimonas arvi*	CC-CFT501	Contig
GCF_004016515.1	x	*Rubrivivax albus*	ICH-3	Contig
GCF_004004565.1	x	*Inhella crocodyli*	CCP-18	Contig
GCF_902459735.1	x	*Pandoraea capi*	LMG 20602	Contig
GCF_902459805.1	x	*Pandoraea bronchicola*	LMG 20603	Contig
GCF_902459685.1	x	*Pandoraea iniqua*	LMG 31009	Contig

Assembly accession	Representative genome	Organism name	Type strain name	Assembly level
GCF_902459615.1	x	*Pandoraea commovens*	LMG 31010	Contig
GCF_902459565.1	x	*Pandoraea aquatica*	LMG 31011	Contig
GCF_902459725.1	x	*Pandoraea eparura*	LMG 31012	Contig
GCF_902459705.1	x	*Pandoraea terrigena*	LMG 31013	Contig
GCF_902459595.1	x	*Pandoraea soli*	LMG 31014	Contig
GCF_902459665.1	na	*Pandoraea cepalis*	LMG 31106	Contig
GCF_902459655.1	x	*Pandoraea anhela*	LMG 31108	Contig
GCF_902459585.1	x	*Pandoraea nosoerga*	LMG 31109	Contig
GCF_902459745.1	x	*Pandoraea communis*	LMG 31110	Contig
GCF_902459555.1	x	*Pandoraea horticolens*	LMG 31112	Contig
GCF_902459645.1	x	*Pandoraea pneumonica*	LMG 31114	Contig
GCF_902459575.1	x	*Pandoraea morbifera*	LMG 31116	Contig
GCF_902459765.1	x	*Pandoraea anapnoica*	LMG 31117	Contig
GCF_902459775.1	x	*Pandoraea captiosa*	LMG 31118	Contig
GCF_006970865.1	x	*Rhodoferax sediminis*	CHu59-6-5	Complete Genome
GCF_009866805.1	x	*Achromobacter aestuarii*	KS-M25	Contig
GCF_006974105.1	x	*Rhodoferax aquaticus*	Gr-4	Complete Genome
GCF_003261235.1	na	*Polynucleobacter paneuropaeus*	MG-25-Pas1-D2	Chromosome
GCF_010078235.1	x	*Undibacterium crateris*	B2R-29	Scaffold
GCF_004310865.1	x	*Aquabacterium lacunae*	KMB7	Contig
GCF_004353915.1	x	*Paraburkholderia silviterrae*	4M-K11	Scaffold
GCF_004681975.1	x	*Ramlibacter humi*	18x22-1	Contig
GCF_004353905.1	x	*Paraburkholderia guartelaensis*	CNPSo 3008	Contig
GCF_004359985.1	x	*Paraburkholderia flava*	LD6	Contig
GCF_004524855.1	x	*Paraburkholderia pallida*	7MH5	Chromosome
GCF_004614185.1	x	*Massilia arenosa*	MC02	Contig
GCF_004614165.1	x	*Duganella callida*	DN04	Contig

Assembly accession	Representative genome	Organism name	Type strain name	Assembly level
GCF_004614195.1	x	*Massilia horti*	ONC3	Contig
GCF_004803635.1	x	*Lampropedia aestuarii*	YIM MLB12	Contig
GCF_006386545.1	x	*Pseudorivibacter rhizosphaerae*	C1-9	Contig
GCF_005871185.1	x	*Limnobacter alexandrii*	LZ-4	Scaffold
GCF_009789655.1	x	*Paraburkholderia acidiphila*	7Q-K02	Complete Genome
GCF_009789675.1	x	*Paraburkholderia acidisoli*	DHF22	Complete Genome
GCF_005876985.1	x	*Rhodoferax bucti*	GSA243-2	Contig
GCF_019343455.1	x	*Alcaligenes ammonioxydans*	HO-1	Complete Genome
GCF_006337085.1	x	*Sutterella faecalis*	KGMB03119	Complete Genome
GCF_008929045.1	x	*Noviherbaspirillum aerium*	122213-3	Scaffold
GCF_007556605.1	x	*Tepidimonas sediminis*	YIM 72259	Contig
GCF_007556595.1	x	*Tepidimonas alkaliphilus*	YIM 72238	Contig
GCF_009760915.1	x	*Azohydromonas aeria*	CFCC 13393	Scaffold
GCF_007655255.1	x	*Extensimonas perlucida*	HX2-24	Scaffold
GCF_006517255.1	x	*Janthinobacterium tructae*	SNU WT1	Complete Genome
GCF_009497155.1	x	*Glaciimonas soli*	GS1	Scaffold
GCF_007431345.1	x	*Chitinimonas arctica*	R3-44	Complete Genome
GCF_009690905.1	na	*Paraburkholderia madseniana*	RP11	Scaffold
GCF_008014745.1	x	*Massilia arenae*	GEM5	Contig
GCF_008369935.1	x	*Paraburkholderia panacisoli*	DCY113	Scaffold
GCF_008831265.1	x	*Lacisediminimonas profundi*	CHu64-6-4	Scaffold
GCF_012844455.2	x	*Comamonas suwonensis*	EJ-4	Contig
GCF_012184415.1	x	*Ramlibacter lithotrophicus*	RBP-1	Contig
GCF_011682065.1	x	*Massilia rubra*	CCM 8692	Scaffold
GCF_008632125.1	x	*Cupriavidus cauae*	MKL-01	Scaffold
GCF_011682175.1	x	*Massilia frigida*	CCM 8695	Contig
GCF_011682145.1	x	*Massilia mucilaginosa*	CCM 8733	Scaffold

Assembly accession	Representative genome	Organism name	Type strain name	Assembly level
GCF_013387465.1	x	*Hydrogenophaga aromaticivorans*	D2P1	Scaffold
GCF_022870505.1	x	*Burkholderia perseverans*	INN12	Chromosome
GCF_009833965.1	x	*Pelistega ratti*	NLN63	Complete Genome
GCF_009208555.1	x	*Janthinobacterium violaceinigrum*	FT13W	Scaffold
GCF_009362785.1	na	*Paraburkholderia atlantica*	CNPSo 3155	Contig
GCF_009362735.1	x	*Paraburkholderia franconis*	CNPSo 3157	Contig
GCF_009857595.1	na	*'Massilia aquatica' Lu et al. 2020*	FT127W	Contig
GCF_009674495.1	x	*Duganella alba*	FT9W	Contig
GCF_009674565.1	x	*Duganella aquatilis*	FT26W	Contig
GCF_009674535.1	x	*Duganella guangzhouensis*	FT80W	Contig
GCF_009674485.1	x	*Massilia rivuli*	FT92W	Scaffold
GCF_003353055.1	x	*Cupriavidus lacunae*	S23	Contig
GCF_013177735.1	x	*Paraburkholderia elongata*	5N	Contig
GCF_013177695.1	x	*Paraburkholderia solitsugae*	1N	Contig
GCF_009861485.1	x	*Massilia puerhi*	SJY3	Contig
GCF_009758015.1	x	*Ramlibacter pinisoli*	MAH-25	Scaffold
GCF_009857605.1	x	*Duganella levis*	CY42W	Contig
GCF_009857535.1	x	*Duganella margarita*	FT109W	Contig
GCF_009857505.1	na	*Duganella lactea*	FT50W	Contig
GCF_009857445.1	x	*Duganella flavida*	FT135W	Scaffold
GCF_009857835.1	x	*Duganella fentianensis*	FT93W	Scaffold
GCF_009857475.1	x	*Massilia guangdongensis*	DS3	Contig
GCF_014237865.1	x	*Pusillimonas minor*	YC-7-48	Contig
GCF_009906855.1	x	*Xylophilus rhododendri*	KACC 21265	Complete Genome
GCF_011090165.1	x	*Duganella aceris*	SAP-35	Scaffold
GCF_013432195.1	x	*Tepidicella baoligensis*	B18-50	Scaffold
GCF_010499455.1	x	*Ideonella livida*	TBM-1	Contig

Assembly accession	Representative genome	Organism name	Type strain name	Assembly level
GCF_010499255.1	x	*Orrella amnicola*	NBD-18	Contig
GCF_010974945.1	x	*Noviherbaspirillum galbum*	17J57-3	Contig
GCF_013003915.1	x	*Massilia aromaticivorans*	ML15P13	Scaffold
GCF_012927045.1	x	*Azohydromonas caseinilytica*	G-1-1-14	Scaffold
GCF_012927085.1	x	*Ramlibacter agri*	G-1-2-2	Contig
GCF_012927125.1	x	*Paraburkholderia antibiotica*	G-4-1-8	Scaffold
GCF_012927275.1	x	*Massilia polaris*	RP-1-19	Scaffold
GCF_012927345.1	x	*Paraburkholderia polaris*	RP-4-7	Contig
GCF_013266755.1	x	*Aquabacterium terrae*	S2	Scaffold
GCF_013155545.1	x	*Caenimonas soli*	S4	Contig
GCF_013366375.1	x	*Schlegelella koreensis*	ID0723	Contig
GCF_009380215.1	x	*Rugamonas aquatica*	FT29W	Scaffold
GCF_009380165.1	x	*Rugamonas rivuli*	FT103W	Scaffold
GCF_013363755.1	x	*Acidovorax antarcticus*	16-35-5	Complete Genome
GCF_014145335.1	x	*Aquariibacter albus*	SJAQ100	Scaffold
GCF_014836675.1	na	*Comamonas avium*	Sa2CVA6	Scaffold
GCF_014284365.1	x	*Undibacterium rugosum*	CY7W	Contig
GCF_014284335.1	x	*Undibacterium hunanense*	CY18W	Scaffold
GCF_014284235.1	x	*Undibacterium curvum*	CY22W	Scaffold
GCF_014284195.1	x	*Undibacterium griseum*	FT31W	Scaffold
GCF_014284175.1	x	*Undibacterium flavidum*	LX15W	Scaffold
GCF_014284155.1	x	*Undibacterium nitidum*	LX22W	Scaffold
GCF_014284125.1	x	*Undibacterium umbellatum*	NL8W	Scaffold
GCF_015689335.1	x	*Massilia antarctica*	P9640	Complete Genome
GCF_014837235.1	x	*Limnohabitans radicicola*	JUR4	Scaffold
GCF_907164555.1	x	*Paraburkholderia saeva*	LMG 31841	Contig
GCF_015159745.1	x	*Ramlibacter aquaticus*	LMG 30558	Contig

Assembly accession	Representative genome	Organism name	Type strain name	Assembly level
GCF_013366925.1	x	*Paraburkholderia youngii*	JPY169	Contig
GCF_015354245.1	na	*Diaphorobacter caeni*	NR2-3-3-1	Contig
GCF_016093545.1	x	*Massilia rhizosphaerae*	NEAU-GH312	Scaffold
GCF_016809835.1	x	*Massilia soli*	R798	Contig
GCF_905220705.1	na	*Paraburkholderia domus*	LMG 31832	Contig
GCF_905221015.1	na	*Paraburkholderia nemoris*	LMG 31836	Contig
GCF_905220975.1	x	*Paraburkholderia haematera*	LMG 31837	Contig
GCF_016093275.1	x	*Inhella proteolytica*	1Y17	Contig
GCF_016093295.1	x	*Inhella gelatinilytica*	4Y10	Contig
GCF_016641735.1	x	*Ramlibacter algicola*	CrO1	Contig
GCF_019186805.1	x	*Comamonas fluminis*	CJ34	Complete Genome
GCF_016595155.1	x	*Advenella mandrilli*	WQ 585	Contig
GCF_016632335.1	x	*Noviherbaspirillum pedocola*	DKR-6	Scaffold
GCF_017498525.1	na	*Ottowia testudinis*	27C	Complete Genome
GCF_907164575.1	na	*Paraburkholderia gardini*	LMG 32171	Contig
GCF_018305005.1	x	*Collimonas silvisoli*	RXD178	Contig
GCF_018304945.1	x	*Collimonas humicola*	RLT1W51	Contig
GCF_018139545.1	x	*Undibacterium fentianense*	FT137W	Scaffold
GCF_018139565.1	x	*Undibacterium rivi*	FT147W	Contig
GCF_018139645.1	x	*Undibacterium baiyunense*	BYS107W	Scaffold
GCF_018139685.1	x	*Undibacterium luofuense*	LFS511W	Contig
GCF_019656455.1	x	*Noviherbaspirillum aridicola*	NCCP-691	Scaffold
GCF_022230885.1	x	*Paenalcaligenes niemegkensis*	NGK35	Contig
GCF_002761755.1	x	*Mitsuaria chitinivorans*	HWN-4	Contig
