# Supplementary material for: Genome-based taxonomy of Burkholderia sensu lato: Distinguishing closely related species
Source: Genet Mol Biol. 2023 Nov 3;46(3 Suppl 1):e20230122. doi: 10.1590/1678-4685-GMB-2023-0122 (PMC10629849; doi:10.1590/1678-4685-GMB-2023-0122)
Supplement: Table S2 - [file 1415-4757-GMB-46-3-s1-e20230122-s7.pdf]

Genetics and Molecular Biology
https://doi.org/10.1590/1678-4685-GMB-2023-0122

Supplementary Material to "Genome-based taxonomy of *Burkholderia sensu lato*: distinguishing closely related species"

Table S2 - List of selected *Burkholderia senso latu* genomes used in this work together with quality features and reclassifications.

Strains analysed in this work*	NCBI				CheckM		Correct species	Code according to Bach et al., 2022
	Acession number	Assembly level	Size (bp)	GC content	Completeness (%)	Contamination (%)		
Burkholderia cepacia ATCC 25416	GCF_000473485.1	Complete	8574389	66.57	99.95	0.13	*Burkholderia cepacia*	
Burkholderia cepacia ATCC 17759	GCA_002906895.1	Contig	8721280	66.4	98.76	0.36	*Burkholderia cepacia*	
Burkholderia cepacia BC16	GCA_009586235.1	Complete	8366868	66.76	99.95	0.13	*Burkholderia cepacia*	
Burkholderia cepacia INT3-BP177	GCA_001718395.1	Complete	7337382	66.8	99.95	0.4	*Burkholderia cepacia*	
Burkholderia cepacia PT02	GCF_003268125.1	Contig	8439210	66.9	99.95	0.13	*Burkholderia cepacia*	
"Burkholderia reimsis" BE51	GCF_003294055.1	Contig	8933940	66.4	99.95	0.29	*Burkholderia cepacia*	
Burkholderia cenocepacia NCTC13227	GCA_900446215.1	Contig	8038740	66.9	99.75	1.26	*Burkholderia cenocepacia*	
Burkholderia cenocepacia H111	GCA_000236215.4	Complete	7714893	67.3	99.65	0.4	*Burkholderia cenocepacia*	
Burkholderia cenocepacia J2315	GCA_000009485.1	Complete	8055782	66.9	99.75	2.59	*Burkholderia cenocepacia*	
Burkholderia cenocepacia MSMB384WGS	GCA_001718895.1	Complete	7780598	67.3	99.75	0.4	*Burkholderia cenocepacia*	
Burkholderia cenocepacia MINF4A	GCA_904865725.1	Complete	7944010	67.11	99.75	0.07	*Burkholderia cenocepacia*	
Burkholderia cenocepacia ST32	GCA_001484665.1	Complete	8090388	67	99.75	0.4	*Burkholderia cenocepacia*	
Burkholderia cenocepacia VC12308	GCA_001999885.1	Complete	7634498	67.2	99.75	0.4	*Burkholderia cenocepacia*	
Burkholderia orbicola Tatl-371	GCF_900100915.1	Contig	7496110	67	99.95	0	*Burkholderia orbicola*	BCC 05
Burkholderia sp. CR318	GCA_002007585.1	Complete	7664894	66.8	99.95	0	*Burkholderia orbicola*	BCC 05

Strains analysed in this work*	NCBI				CheckM		Correct species	Code according to Bach et al., 2022
	Acession number	Assembly level	Size (bp)	GC content	Completeness (%)	Contamination (%)		
Burkholderia sp. MC0-3	GCA_000019505.1	Complete	7971389	66.6	99.95	0	*Burkholderia orbicola*	BCC 05
Burkholderia sp. FL-5-3-30-S1-D7	GCA_001718515.1	Complete	6330751	67.1	99.95	0	*Burkholderia orbicola*	BCC 05
Burkholderia sp. VC12802	GCA_001999825.1	Complete	7394909	67.1	99.95	0	*Burkholderia orbicola*	BCC 05
Burkholderia sp. VC7848	GCA_001999785.1	Complete	7499459	66.9	99.95	0.4	*Burkholderia orbicola*	BCC 05
Burkholderia sp. PC184Mulks	GCF_003076415.1	Complete	7067050	66.83	99.95	0	*Burkholderia orbicola*	BCC 05
Burkholderia sp. HI2424	GCF_000203955.1	Complete	7702840	66.8	99.95	0	*Burkholderia orbicola*	BCC 05
"Burkholderia semiarida" CCRMBC74	GCF_029268935.1	Scaffold	7577000	66.5	99.75	0.23	*"Burkholderia semiarida"*	BCC 03
"Burkholderia semiarida" CCRMBC171	GCF_029268915.1	Scaffold	7547000	67	99.75	0.49	*"Burkholderia semiarida"*	BCC 03
"Burkholderia semiarida" CCRMBC16	GCF_029268975.1	Scaffold	7529000	67	99.95	0.23	*"Burkholderia semiarida"*	BCC 03
"Burkholderia semiarida" CCRMBC33	GCF_029269015.1	Scaffold	7542000	67	99.55	1.13	*"Burkholderia semiarida"*	BCC 03
Burkholderia sp AZ4-2-10-S1D7	GCA_001547525.1	Chromosome	7273051	67.2	99.75	0.93	*"Burkholderia semiarida"*	BCC 03
Burkholderia sp XXVI	GCA_002925705.1	Scaffold	7692989	67	99.75	1.23	*"Burkholderia semiarida"*	BCC 03
"Burkholderia sola" CCRMBC51	GCF_029268985.1	Scaffold	8184000	66.5	99.35	1.26	*"Burkholderia sola"*	
Burkholderia sp. YG-3	GCA_003966315.1	Complete	8036463	66.8	99.75	1.43	new species	BCC 06
Burkholderia sp. Bu72	GCA_000504665.1	Scaffold	7423806	66.9	99.75	0.4	new species	BCC 07
Burkholderia sp. DWS 37UF10B-2	GCA_000756845.1	Scaffold	7182132	67.2	99.75	0.8	new species	BCC 09
Burkholderia sp. CEIB S5-2	GCF_001541445.1	Contig	8976050	65.7	99.75	6.09	new species	
Burkholderia sp. Bp9038	GCA_003858255.1	Contig	7637496	66.8	99.95	0.83	new species	
Burkholderia sp. Bp8974	GCA_003854775.1	Contig	7760805	66.7	99.95	1.23	new species	
Burkholderia sp. BCC0506	GCF_902833145.1	Scaffold	7300000	67	99.75	0.49	new species	

Strains analysed in this work*	NCBI				CheckM		Correct species	Code according to Bach et al., 2022
	Acession number	Assembly level	Size (bp)	GC content	Completeness (%)	Contamination (%)		
***Burkholderia territorii* LMG 28158**	GCA_902499035.1	Contig	6907990	66.7	99.95	0.4	*Burkholderia territorii*	
Burkholderia territorii MSMB2203WGS	GCA_001636095.1	Chromosome	6883104	66.5	99.95	0.1	*Burkholderia territorii*	
Burkholderia territorii RF8-non-BP5	GCA_001718335.1	Complete	6902370	66.7	99.95	0.4	*Burkholderia territorii*	
"Burkholderia paludis" Msh1	GCA 000732615.1	Contig	8633651	67.1	99.95	1.69		
***Burkholderia mallei* ATCC 23344**	GCF_000011705.1	Complete	5835530	68.51	99.95	0	*Burkholderia mallei*	
***Burkholderia pseudomallei* ATCC 23343**	GCF_001182285.1	Scaffold	7037420	68.3	99.72	0	*Burkholderia pseudomallei*	
***Burkholderia oklahomensis* C6786**	GCF_001522135.2	Complete	7134790	67	99.95	0.91	*Burkholderia oklahomensis*	
"Burkholderia mayonis" BDU6	GCF_001523745.2	Complete	6590910	66.27	99.95	0.15	*"Burkholderia mayonis"*	
***Burkholderia plantarii* ATCC 43733**	GCF_001411805.1	Complete	8081050	68.5	99.95	1.59	*Burkholderia plantarii*	
"Burkholderia perseverans" INN12	GCF_022870505.1	Chromosome	7668750	68.9	99.95	1.06	*"Burkholderia perseverans"*	
***Caballeronia humi* LMG 22934**	GCF 001544475.1	Contig	7619203	63.3	99.95	0.51	*Caballeronia humi*	
***Caballeronia humi* KEMC 7302-068**	GCF_007474635.1	Contig	7148193	63.3	99.95	1.23	*Caballeronia humi*	
***Caballeronia terrestris* LMG 22937**	GCF 001544515.1	Contig	8201357	63.0	100.00	0.97	*Caballeronia terrestris*	
***Paraburkholderia agricolaris* BaQS159**	GCF_009455635.1	Contig	8721420	61.9	99.95	0.28	*Paraburkholderia agricolaris*	
***Paraburkholderia fungorum* LMG 16225**	GCF_902833645.1	Scaffold	8926561	61.8	99.95	0.66	*Paraburkholderia fungorum*	
Paraburkholderia insulsa LMG 28183	GCF 003002115.1	Contig	9620907	61.6	99.95	2.77	*Paraburkholderia insulsa*	
***Paraburkholderia aspalathi* LMG 27731**	GCF_900116445.1	Scaffold	9892290	61.1	99.55	0.46	*Paraburkholderia aspalathi*	

Strains analysed in this work*	NCBI				CheckM		Correct species	Code according to Bach et al., 2022
	Acession number	Assembly level	Size (bp)	GC content	Completeness (%)	Contamination (%)		
Paraburkholderia nemoris LMG 31836	GCF_905221015.1	Contig	9274800	61.6	99.95	0.8	*Paraburkholderia nemoris*	
"Paraburkholderia atlantica" CCGE1002	GCF_000092885.1	Complete	7884860	63.2	99.95	0	*"Paraburkholderia atlantica"*	
Paraburkholderia youngii JPY169	GCF_013366925.1	Contig	9492390	62.8	99.95	0.58	*Paraburkholderia youngii*	
"Paraburkholderia caffeinitolerans" LMG 28688	GCF 902859945.1	Contig	8054104	64.1	99.95	0.92	*"Paraburkholderia caffeinitolerans"*	
"Paraburkholderia dokdonella" DCR-13	GCF_003286395.1	Complete	4404244	64.1	97.16	0.73	*"Paraburkholderia dokdonella"*	
Paraburkholderia dioscoreae PDMSB31	GCF_902459535.1	Complete	8347503	62.5	99.87	0.33	*Paraburkholderia dioscoreae*	
Paraburkholderia xenovorans LB400	GCF_000756045.1	Complete	9702950	62.6	99.95	0.42	*Paraburkholderia xenovorans*	
Paraburkholderia pallida 7MH5	GCF_004524855.1	Chromosome	10394431	64.1	99.95	1.13	*Paraburkholderia pallida*	
Paraburkholderia oxyphila NBRC 105797	GCF_000685075.1	Contig	10647665	64.1	99.95	1.52	*Paraburkholderia oxyphila*	
Paraburkholderia silviterrae 4M-K11	GCF_004353915.1	Contig	9169990	64.4	99.95	0.37	*Paraburkholderia silviterrae*	
Paraburkholderia caledonica NBRC102488	GCF 000685095.1	Contig	7282355	62	99.95	0.03	*Paraburkholderia caledonica*	
Paraburkholderia strydomiana WK1_1f	GCF 004334935.1	Contig	8397958	61.3	93.16	0.6	*Paraburkholderia strydomiana*	

Strains analysed in this work*	NCBI				CheckM		Correct species	Code according to Bach et al., 2022
	Acession number	Assembly level	Size (bp)	GC content	Completeness (%)	Contamination (%)		
Paraburkholderia dipogonis ICMP19430	GCF 004402975.1	Contig	10263517	61.9	99.91	1.83	*Paraburkholderia dipogonis*	
Paraburkholderia phytofirmans PsJN	GCF 000020125.1	Complete	8214658	62.6	99.95	0.40	*Paraburkholderia phytofirmans*	
Paraburkholderia steynii Hb1_1ba	GCF 004334975.1	Contig	11448031	61.8	91.68	0.96	*Paraburkholderia. steynii*	
Paraburkholderia steynii YR281	GCF_900100565.1	Scaffold	10306168	61.5	99.31	0.57	*Paraburkholderia terrae*	
Paraburkholderia terrae DSM 17804	GCF 002902925.1	Complete	10062489	62.3	99.95	0.38	*Paraburkholderia terrae*	
Paraburkholderia terrae KU-64	GCF_020885575.1	Complete	10400000	61.5	99.5	1.3	*Paraburkholderia terrae*	
Paraburkholderia terrae KU-15	GCF_023169865.1	Complete	10400000	61.5	99.81	2.34	*Paraburkholderia terrae*	
Paraburkholderia hospita DSM 17164	GCF 002902965.1	Complete	11527706	62.2	99.6	0.38	*Paraburkholderia hospita*	
Paraburkholderia hospita LMG 20598	GCF_902833685.1	Scaffold	11300000	61.5	99.53	0.24	*Paraburkholderia hospita*	
Paraburkholderia hospita BS001	GCF_000265115.1	Contig	11300000	61.5	99.5	1.98	*Paraburkholderia hospita*	
Paraburkholderia hospita BS007	GCF_002157295.1	Contig	11000000	61.5	99.53	0.94	*Paraburkholderia hospita*	
Paraburkholderia hospita BS110	GCF_002157285.1	Contig	11200000	61.5	99.69	1.83	*Paraburkholderia hospita*	
Paraburkholderia hospita BS437	GCF_002157305.1	Contig	11300000	61.5	99.21	0.54	*Paraburkholderia hospita*	
Paraburkholderia hospita mHSR1	GCF_003330805.1	Complete	10800000	61.5	99.53	0.09	*Paraburkholderia hospita*	
Paraburkholderia hospita LMG 20598	GCF_900108355.1	Scaffold	11200000	61.5	99.27	0.26	*Paraburkholderia hospita*	
Paraburkholderia hospita BT03	GCF_900167965.1	Contig	11000000	61.5	99.75	0.12	*Paraburkholderia hospita*	
Paraburkholderia hospita YR277	GCF_900168175.1	Contig	10800000	61.5	99.72	0.24	*Paraburkholderia hospita*	
***Paraburkholderia caribensis* DSM 13236**	GCF_002902945.1	Complete	9000000	62.5	99.75	1.28	*Paraburkholderia caribensis*	
Paraburkholderia caribensis MWAP64	GCF_001449005.1	Complete	9000000	62.5	99.04	1.28	*Paraburkholderia caribensis*	
Paraburkholderia caribensis Bcrs1W	GCF_001611015.1	Complete	9300000	62	99.75	1.04	*Paraburkholderia caribensis*	
Paraburkholderia caribensis TJ182	GCF_003028645.1	Contig	9200000	62	99.75	1.43	*Paraburkholderia caribensis*	
Paraburkholderia caribensis 852011	GCF_013378095.1	Complete	8500000	62.5	99.94	1.21	*Paraburkholderia caribensis*	
Paraburkholderia caribensis IAC-BECa88	GCF_020544345.1	Complete	8400000	62.5	97.14	0.09	*Paraburkholderia caribensis*	

Strains analysed in this work*	NCBI				CheckM		Correct species	Code according to Bach et al., 2022
	Acession number	Assembly level	Size (bp)	GC content	Completeness (%)	Contamination (%)		
Paraburkholderia caribensis SKND8	GCF_023952555.1	Scaffold	9200000	62	99.75	1.43	*Paraburkholderia caribensis*	
Paraburkholderia caribensis LMG 18531	GCF_902833515.1	Scaffold	9000000	62.5	99.94	1.28	*Paraburkholderia caribensis*	
Paraburkholderia caribensis BCC1824	GCF_902833585.1	Contig	9000000	62.5	99.5	0.97	*Paraburkholderia caribensis*	
Paraburkholderia caribensis PCAR1334	GCF_914484885.1	Scaffold	8700000	62.5	98.16	1.65	*Paraburkholderia caribensis*	
Paraburkholderia caribensis MBA4	GCF_000522545.2	Complete	9500000	62	99.5	0.2	*Paraburkholderia caribensis*	
Paraburkholderia caribensis PCAR477	GCF_914492705.1	Scaffold	9800000	62	99.75	1.21	new species	
Paraburkholderia terrae 19C8	GCF_022179825.1	Contig	9300000	62.5	99.75	0.83	new species	
Paraburkholderia phymatum STM815	GCF_000020045.1	Complete	8700000	62	99.75	1.21	*Paraburkholderia phymatum*	
Paraburkholderia azotifigens NF2-5-3	GCF_007995085.1	Contig	9700000	62	99.75	0.54	*Paraburkholderia azotifigens*	
Paraburkholderia sabiae LMG24235	GCF_904848645.1	Contig	9700000	62	99.31	0.4	*Paraburkholderia sabiae*	
Paraburkholderia sabiae PSAB630	GCF_914484815.1	Scaffold	8300000	62	99.94	0.31	*Paraburkholderia sabiae*	
Paraburkholderia diazotrophica LMG26031	GCF_900108945.1	Scaffold	8700000	62.5	99.94	0.83	*Paraburkholderia diazotrophica*	
Paraburkholderia franconis CNPSo 3157	GCF_009362735.1	Contig	10000000	62	97.39	1.73	*Paraburkholderia franconis*	
Paraburkholderia piptadeniae STM7183	GCF_900007165.1	Scaffold	9700000	62	99.94	0.98	*Paraburkholderia piptadeniae*	
"Paraburkholderia bonniae" BbQS859	GCF 009455625.1	Contig	4098182	58.8	99.46	0.4	*"Paraburkholderia bonniae"*	
"Paraburkholderia hayleyella" BhQS11	GCF 009455685.1	Contig	4125700	59.3	99.42	0.40	*"Paraburkholderia hayleyella"*	

*Type strains are shown in bold.
