# Supplementary material for: Genome-based taxonomy of Burkholderia sensu lato: Distinguishing closely related species
Source: Genet Mol Biol. 2023 Nov 3;46(3 Suppl 1):e20230122. doi: 10.1590/1678-4685-GMB-2023-0122 (PMC10629849; doi:10.1590/1678-4685-GMB-2023-0122)
Supplement: Table S3 - [file 1415-4757-GMB-46-3-s1-e20230122-s8.pdf]

Genetics and Molecular Biology
https://doi.org/10.1590/1678-4685-GMB-2023-0122

Supplementary Material to "Genome-based taxonomy of *Burkholderia sensu lato*: distinguishing closely related species"

Table S3 - Clusters of identity/similarity formed by the *Burkholderia sensu latu* genomes analysed in this work using FastANI coupled with ProKlust.

Cluster	Species name	Genome accession
1	*Burkholderia cepacia ATCC 25416*	GCF_000473485.1
1	*Burkholderia cepacia ATCC 25416 UCB 717*	GCF_001411495.1
1	*Burkholderia cepacia NBRC 14074*	GCF_001528845.1
1	*Burkholderia reimsis BE51*	GCF_003294055.1
1	*Burkholderia cepacia ATCC 25416*	GCF_003546465.1
1	*Burkholderia cepacia ATCC 25416*	GCF_006094315.1
1	*Burkholderia cepacia NCTC10743*	GCF_900446175.1
2	*Burkholderia mallei ATCC 23344*	GCF_000011705.1
2	*Burkholderia pseudomallei ATCC 23343*	GCF_001182285.1
2	*Burkholderia pseudomallei WRAIR 286*	GCF_006538545.1
2	*Burkholderia mallei NCTC12938*	GCF_900446245.1
3	*Burkholderia oklahomensis C6786*	GCF_000170375.1
3	*Burkholderia oklahomensis C6786*	GCF_000959365.1
3	*Burkholderia oklahomensis C6786*	GCF_001522135.2
3	*Burkholderia mayonis BDU6*	GCF_001523745.2
3	*Burkholderia oklahomensis LMG 23618*	GCF_900608545.1
3	*Burkholderia oklahomensis LMG 23618*	GCF_902829515.1
4	*Burkholderia plantarii ATCC 43733*	GCF_001411805.1
4	*Burkholderia perseverans INN12*	GCF_022870505.1
4	*Burkholderia plantarii LMG 9035*	GCF_902832905.1
5	*Paraburkholderia aspalathi LMG 27731*	GCF_900116445.1
5	*Paraburkholderia nemoris LMG 31836*	GCF_905221015.1
6	*Paraburkholderia dokdonella DCT13*	GCF_003286395.1
6	*Paraburkholderia caffeinitolerans LMG 28688*	GCF_902859945.1
7	*Paraburkholderia phytofirmans PsJN*	GCF_000020125.1
7	*Paraburkholderia dipogonis ICMP 19430*	GCF_004402975.1
8	*Paraburkholderia insulsa LMG 28183*	GCF_003002115.1
8	*Paraburkholderia agricolaris BaQS159*	GCF_009455635.1
8	*Paraburkholderia fungorum LMG 16225*	GCF_902833645.1
9	*Paraburkholderia oxyphila NBRC 105797*	GCF_000685075.1
9	*Paraburkholderia pallida 7MH5*	GCF_004524855.1
10	*Paraburkholderia terrae NBRC 100964*	GCF_000739835.1
10	*Paraburkholderia terrae DSM 17804*	GCF_002902925.1

Cluster	Species name	Genome accession
10	*Paraburkholderia hospita DSM 17164*	GCF_002902965.1
10	*Paraburkholderia hospita LMG 20598*	GCF_900108355.1
10	*Paraburkholderia hospita LMG 20598*	GCF_902833685.1
